# Hormonal Contraception and Bone Metabolism: Emerging Evidence from a Systematic Review and Meta-Analysis of Studies on Post-Pubertal and Reproductive-Age Women

**DOI:** 10.3390/ph18010061

**Published:** 2025-01-08

**Authors:** Alice Tassi, Ambrogio P Londero, Anjeza Xholli, Giulia Lanzolla, Serena Bertozzi, Luca Savelli, Federico Prefumo, Angelo Cagnacci

**Affiliations:** 1 Obstetrics and Gynecology Unit, Morgagni-Pierantoni Hospital, 47121 Forlì, Italy; alicetassi9@gmail.com (A.T.);; 2 Department of Neuroscience, Rehabilitation, Ophthalmology, Genetics, Maternal and Infant Health, University of Genoa, 16132 Genova, Italy; 3 Obstetrics and Gynecology Unit, IRCCS Istituto Giannina Gaslini, 16147 Genova, Italy; federicoprefumo@gaslini.org; 4 Academic Unit of Obstetrics and Gynecology, IRCCS Ospedale San Martino, 16132 Genoa, Italy; 5 Department of Clinical and Experimental Medicine, Endocrinology Unit II, University of Pisa and University Hospital of Pisa, 56126 Pisa, Italy; 6 Department of Orthopaedic Surgery, Perelman School of Medicine, University of Pennsylvania, Philadelphia, PA 19104, USA; 7 Breast Unit, University Hospital of Udine, 33100 Udine, Italy; 8 Dipartimento di Scienze Mediche e Chirurgiche (DIMEC), University of Bologna, 40138 Bologna, Italy

**Keywords:** estrogens, progestins, bone turnover, osteocalcin, standardized mean change, progestins, androgenic effect

## Abstract

Background/Objectives: This study aims to assess the effects of combined hormonal contraceptives (CHCs) on bone metabolism markers. It primarily measures osteocalcin and additionally examines other bone health markers, seeking to determine their responses to estrogen–progestogen treatments. Methods: This study involved a comprehensive evaluation of the pertinent literature and a meta-analysis explicitly conducted on data describing women of reproductive age. The analysis encompassed accessible papers ranging to December 2024 (i.e., those listed in PubMed/Medline, Embase, Scopus, the Cochrane Database, International Clinical Trials Registry, and ClinicalTrials.gov). We examined published randomized controlled trials (RCTs) and prospective studies. The quality of the studies was assessed using the Cochrane tool for RCTs and the Newcastle–Ottawa Scale for prospective studies. The selected indicators for primary and secondary outcomes were ascertained by standardized mean change (SMC), displaying the difference between conditions before and after treatment. Trends were evaluated using meta-regressions. Results: Ultimately, 34 articles out of 1924 identified items met the inclusion criteria, covering 33 unique studies. In EE/E4 combinations, osteocalcin dropped significantly (SMC −0.54 (CI.95 −0.64/−0.43) and −0.43 (CI.95 −0.76/−0.10)). Similar effects were observed for other bone-formation and reabsorption markers, with less significant reductions observed in E2-containing CHC (e.g., alkaline phosphatase (bone) EE combinations, SMC −0.39 (CI.95 −0.67/−0.11); P1NP E2 combination, 0.12 (CI.95 −0.10/0.33); and EE combinations, −0.55 (CI.95 −0.83/−0.26)). The reduction patterns also exhibited differences according to the women’s age (e.g., osteocalcin in EE combinations ≤21, SMC −0.63 (CI.95 −0.77/−0.49) and >21, SMC −0.42 (CI.95 −0.61/−0.24); alkaline phosphatase (bone) EE combinations ≤21, SMC −0.55 (CI.95 −0.86/−0.24) and >21, SMC −0.06 (CI.95 −0.47/0.35)). This analysis found that CHC maintains or reduces bone turnover in childbearing women, with effects varying by age and hormone combination. Moreover, bone-formation and reabsorption markers correlated positively to pro-androgenic progestins (*p* < 0.05). Thus, estrogen–progestogen combinations reduce bone turnover less when weak estrogens and a pro-androgenic or neutral progestin are present. Conclusions: This study found that CHCs reduce bone turnover, with natural estrogens and androgenic progestins appearing to be more beneficial than EE and anti-androgenic types. These findings would potentially influence decisions relevant to CHC prescriptions during a woman’s reproductive phases, emphasizing the need for additional research to tailor CHC usage to bone health.

## 1. Introduction

Combined hormonal contraceptives (CHCs) are widely used in adolescents and adult women to prevent pregnancy and to treat several gynecological and endocrinological disorders [1,2]. They are often considered a single class of compounds, but in fact, they are a combination of different estrogens, particularly ethynyl-estradiol (EE), estradiol (E2), or estetrol (E4), and neutral, pro-androgenic, or anti-androgenic progestins [3,4]. Throughout a woman’s life, the sex-steroid environment influences bone metabolism, and similarly, an influence on bone turnover is exerted by modifications of the sex-steroid environment via the administration of CHCs. The effect of this has sometimes been reported to be positive, neutral, or even negative [5,6,7,8,9]. The diverging evidence was probably dependent on the reproductive phase investigated or on the composition of the CHC used. The data rather consistently indicate a bone-sparing effect of CHCs in perimenopausal women, but they are less clear as to young post-pubertal women [5,6,7,8]. Bone-turnover modifications were frequently used, because they show sensitivity in evaluating short-term effects, whereas BMD requires at least several years of continuous use in prospective studies, and fracture modifications are difficult to assess in populations with a relatively low rate of such events [10,11,12].

While the existing evidence suggests that CHCs may influence bone turnover, findings are inconsistent, and the impacts on bone metabolism of administering CHCs with different estrogen molecules and combined with different progestins, as assessed using bone-turnover modifications, is unclear.

This analysis aimed to determine if bone-turnover indices of post-pubertal and reproductive-age women were differently modified by CHCs containing varying estrogenic and progestin molecules (Supplemental Table S1). Understanding these differences is of paramount importance, given the widespread use of CHC and the high impact that osteoporosis has in terms of morbidity and mortality at later ages.

## 2. Results

### 2.1. Study Selection

**Figure 1:**
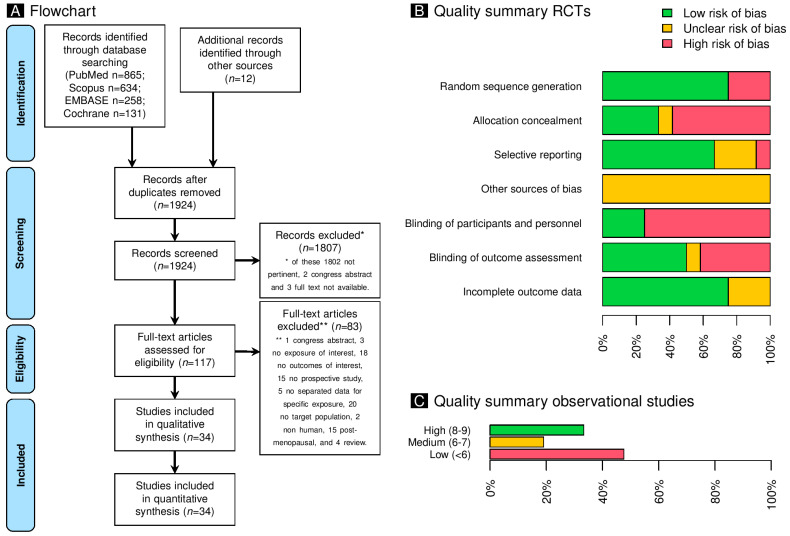
Panel (**A**): This panel shows a graphical representation of the comprehensive study flowchart, which shows the sequential progression of study selection. Panel (**B**): The panel displays a summary of the quality of the randomized clinical trials, using the Cochrane collaboration instrument. Panel (**C**): The panel summarizes the Newcastle–Ottawa Quality Assessment Scale for observational research. The high quality (8–9 points) corresponds to a low risk of bias, and the low quality (<6 points) corresponds to a high risk of bias.

Our literature search, depicted in Figure 1A, initially identified 1924 studies. After screening titles and abstracts, 1807 were excluded for irrelevance, unavailability of full text, or being in congress-abstract form, leaving 117 potentially eligible. Ultimately, 34 articles, covering 33 unique studies, met the inclusion criteria [13,14,15,16,17,18,19,20,21,22,23,24,25,26,27,28,29,30,31,32,33,34,35,36,37,38,39,40,41,42,43,44,45,46]. Orsolini and coworkers and Caldeirão and coworkers present data from the same study, albeit with different bone metabolites being examined [13,44]. Detailed reasons for exclusion are outlined in Figure 1A and Supplemental Table S2.

### 2.2. Study Characteristics

The included studies, conducted from 1968 to 2024, focused on healthy women aged 12–45 and explored different estrogen and progestin types, including ethinylestradiol (EE), estradiol (E2), estradiol valerate (E2V), and estetrol (E4). These studies provided data from 77 research arms, and involving 3757 women. Detailed descriptions are presented in Table 1 and Supplemental Table S3.

### 2.3. Risk of Bias of Included Studies

The research included in the analysis was of variable quality, with 13 randomized trials and 21 prospective cohort or case-control studies. As a result, the varying degrees of quality of the studies included in the analysis are classified as IB, IIB, or IIIB by the CEBM classification [47]. The Cochrane collaboration approach was used to evaluate the randomized studies, and the results are shown in Figure 1B and Supplemental Figure S1A. In the meantime, the remaining potential studies were evaluated using the Newcastle–Ottawa Quality Assessment Scale. Figure 1C and Supplemental Figure S1B show the results of this evaluation.

### 2.4. Bone Markers: Metabolism in Healthy Women

This study aimed to assess the modification of OC, a bone-formation marker, and its association with other related markers (Table 2 and Supplemental Figure S2A). Estrogen-containing contraceptives, notably E4 and EE, significantly reduced OC levels. The control group, without contraceptives or placebo, showed a non-significant OC decrease, similar to the effect of E2V. Progestin-only treatments, however, increased OC levels (*p* < 0.001). Other bone-formation and resorption markers were also reduced when estrogen-type stratification was considered (Table 2 and Supplemental Figures S2A–C, S3A,B, S4A–D). Although the reduction levels of analyzed bone-formation and bone-resorption markers were similar between studies with a maximum age of up to 21 years and those with a maximum age of more than 21 years, some differences emerged in the control groups, as well as some differences in the magnitudes of the standardized effects observed. Table 2 also shows the pooled standardized effects, organized by maximum age.

Bone-formation and bone-resorption marker SMCs were also stratified according to the androgenic effects of progestins, showing higher negative SMCs for anti-androgenic progestins than pro-androgenic and neutral (Table 2).

**Figure 2:**
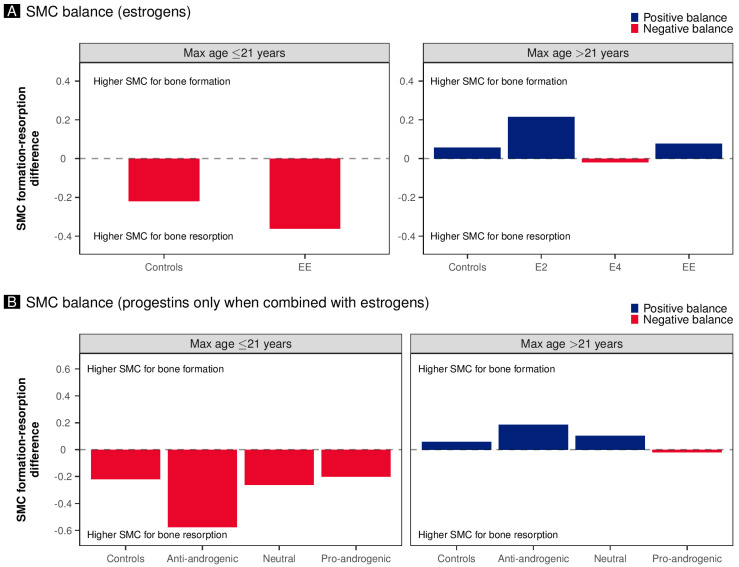
The figures show the balance of bone-formation and bone-resorption markers, expressed as standardized mean change (SMC). Differences were expressed as the difference between the pooled SMCs of all formation markers and the pooled SMCs of all resorption markers. Furthermore, the results are stratified by the maximum age of the women enrolled in the analyzed studies (≤21 vs. >21). Panel (**A**) depicts the balance based on the estrogens used in estrogen–progestin combinations, the controls, and the women treated solely with progestins. The only progestins in the group were LNG and MPA. Panel (**B**) shows the balance of progestins used in the estrogen–progestin medications.

In Table 3, we analyze the stratifications for estrogens and progestins for all formation markers, finding the same patterns overall for the individually analyzed markers. The same evidence emerges for resorption markers and the related stratifications by maximum age. However, the effect on resorption markers is accentuated to a different extent based on the maximum age of the population analyzed. This finding applies both to stratification based on estrogen and that based on the androgenic effects of progestins (Table 3). As a result, Figure 2A,B demonstrate the balance of bone-formation and bone-resorption markers, expressed as the SMC difference. Although the pattern shows a reduction in formation and resorption markers, this reduction affects formation markers differently than resorption markers. In particular, in studies with women with a maximum age of less than 21 years, in the controls, we have a negative balance in favor of resorption markers. In contrast, in studies with women with a maximum age of more than 21, there is a minor positive overall effect in favor of bone formation. We also see how the estrogens (Figure 2A) and progestins associated with the estrogens (Figure 2B) affect the balance compared to the controls.

### 2.5. Meta-Regressions of the Androgenic Effects of Progestins, the Estrogenic Effect on SHBG, and Bone Markers in Healthy Women

**Figure 3:**
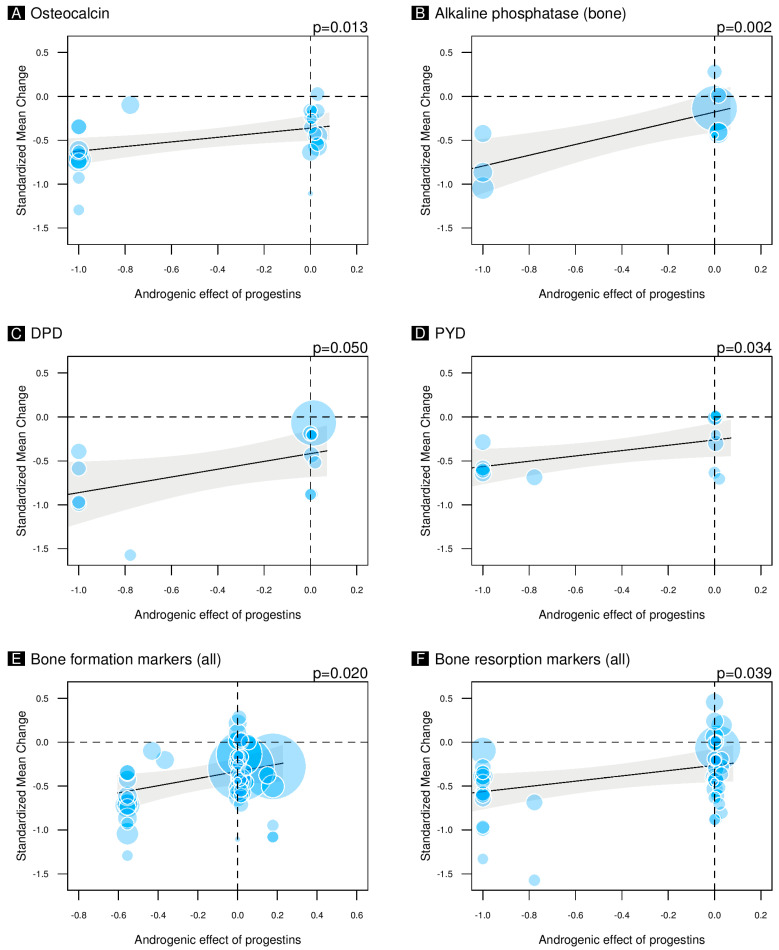
The figures illustrate the results of univariate meta-regressions that examine the androgenic effects of progestins when combined with estrogens in the treatment of healthy women. The evaluation of the androgenic influence was carried out using the following methodology: progestins exhibiting anti-androgenic properties were assigned a numerical value of −1, progestins lacking androgenic effects were assigned a numerical value of 0, and progestins demonstrating pro-androgenic effects were assigned a numerical value of 1. The previously described androgenic effect (−1, 0, or 1) was multiplied by the progestin dose values in micrograms, and the resulting value was then subjected to min–max normalization. Panel (**A**) shows the correlation between the androgenic effects of progestins and the standardized mean change of osteocalcin (still significant after adjustment for estrogens, publication, follow-up length, and maximum age *p* = 0.009). Panel (**B**) shows the correlation between the androgenic effects of progestins and the standardized mean change of alkaline phosphatase (only bone-specific) (not significant in multivariate analysis). Panel (**C**) shows the correlation between the androgenic effects of progestins and the standardized mean change of DPD (not significant in multivariate analysis). Panel (**D**) shows the correlation between the androgenic effects of progestins and the standardized mean change of PYD (not significant in multivariate analysis). Panel (**E**) shows the multivariate correlation (adjustment for estrogens, publication, follow-up length, and maximum age) between the androgenic effects of progestins and the standardized mean change values of all bone-formation markers. Panel (**F**) shows the multivariate correlation (adjustment for estrogens, publication, follow-up length, and maximum age) between the androgenic effects of progestins and the standardized mean change of all bone-resorption markers.

The meta-regressions indicated that anti-androgenic progestins combined with estrogens led to a greater decrease in OC levels (Figure 3A). Bone-specific ALP and resorption markers DPD and PYD also exhibited significant trends (Figure 3B–D). Supplemental Figure S5A,B show the non-significant meta-regressions for ALP (unspecified) and CTX. Moreover, univariate meta-regressions showed a negative correlation between the estrogenic SHBG effect and OC SMC (*p* = 0.029) (Supplemental Figure S6A). Supplemental Figure S6B–E show the non-significant correlations between the estrogenic SHBG effect and other bone markers. Multivariate meta-regressions maintained these trends (Supplemental Figure S7A–F). However, considering the markers independently, the only significant correlation, after adjustment for estrogens, publication, follow-up length, and maximum age, was the correlation between the androgenic effects of progestins and the SMC of OC (Supplemental Figure S7A). Considering all the SMCs of the bone-formation markers in the multivariate analysis, and adjusting for estrogens, publication, follow-up length, and maximum age, the androgenic effects of progestins was still significant (*p* = 0.020) (Figure 3E). Meanwhile, the estrogenic effect on SHBG was not significant (*p* = 0.179). The androgenic effects of progestins was still significant in multivariate analysis for all bone-resorption markers, while the estrogenic effect on SHBG was not significant (*p* = 0.040 and *p* = 0.439, respectively) (Figure 3F).

### 2.6. Calcium Levels and Bone Mineral Density in Healthy Women

Calcium-level changes in urine and serum were generally non-significant (Supplemental Figure S8A,B). BMD analysis found no significant changes in spine and forearm BMD post-contraceptive-use (Supplemental Figure S9A–E). A notable increase in total-body BMD was observed in EE users, as attributed in a study by Orsolini et al. [44]. No significant associations were noted between BMD and androgenic or estrogenic effects (Supplemental Figure S10A–F).

### 2.7. Publication Bias

As shown in Supplemental Figure S11, the publication bias assessment revealed no significant bias for most markers, including OC. However, markers P1NP, DPD, and CTX exhibited potential publication bias (Supplemental Figure S11D,E,G).

## 3. Discussion

### 3.1. Key Results

In this study, bone-turnover markers were investigated. Overall, the meta-analysis indicates that CHC tends to be neutral or to reduce bone turnover in women of childbearing age. However, differences appear based on the age of the women and the estrogen–progestin combination. At all ages, a lower reduction in bone turnover and a possible advantageous balance between formation and absorption is attained by CHC containing weak estrogens and androgenic progestins. Vice versa, a higher reduction in bone turnover and a possible detrimental balance between formation and absorption is induced by CHC containing strong estrogens and anti-androgenic progestins.

### 3.2. Interpretation and Comparison with the Literature

The development of peak bone-mass in adolescence is essential [48,49,50]. Factors that disrupt bone accrual during this period, including the use of hormonal contraceptives, may lead to lifelong consequences, especially if initiated prior to peak bone-mass attainment [51,52]. Adolescents utilizing low-dose CHC may experience diminished peak bone-mass accrual, a condition potentially intensified by decreased IGF-1 production resulting from hepatic estrogen metabolism. Conversely, CHCs may have a bone-preserving effect in adults by suppressing markers of bone turnover [51,53].

#### 3.2.1. Bone Turnover in Women ≤21 Years of Age

Peak bone-mass is crucial in determining the likelihood of developing osteoporosis. During the growth spurt, bone mass and turnover increase in girls, reaching their highest rates between Tanner stages 2 and 3 of puberty [49,50]. Bone accrual is maximum at about 12 years of age, shortly after the highest point of the rate of increase in height [54,55]. In the post-menarche period, the bone mass continues to increase, even though markers of bone metabolism decrease [10,56,57,58]. The timing of peak bone-mass at the hip was reported to be around 16–19 years of age [59], while at the lumbar spine, there was some indication of bone increases up to 33–40 years of age [59]; in other cases, a 1.5% bone decrease at the lumbar spine and distal radius was observed between 20–29 years of age [60].

The 6 months of administration of CHCs did not impact total-body and hip BMD in women 12–22 years of age [61]. Yet a meta-analysis of international prospective controlled studies revealed that CHC users had a substantial reduction in BMD increase compared to non-user adolescents over two years. A recent literature analysis reached a similar conclusion, pointing out the very limited amount of data [62]. Prescription of CHCs is frequently used in adolescents to treat menstrual irregularities, acne, hirsutism, or dysmenorrhea [63], and to define their safe impact on bone is of paramount importance.

In our analysis, control women ≤21 years of age experienced a physiological decrease in bone turnover, which was more evident in bone formation than absorption. This finding is consistent with the deceleration of bone growth in this population of women [10]. The decrease in bone turnover was magnified by CHC containing EE, with a more pronounced effect on bone formation than on absorption. This evidence matches the reported detrimental effect of CHC on peak bone-mass [61,62,64].

Interestingly, the effect of EE was modulated by the associated progestin. The effect was maximal when EE was associated with an anti-androgenic progestin and almost negligible when it was associated with neutral or androgenic progestins. Androgens are known to play a role in bone structure maintenance. Androgen receptors are expressed in osteoblasts and osteoclasts, respectively, and activation of these two receptors was shown to increase bone mass by increasing osteoblast activity and decreasing osteoclastic bone resorption [65]. Likely, the pronounced ovarian suppression induced by EE, the elevated synthesis of free sex-hormone-binding globulin (SHBG) induced by EE, and the anti-androgenic effect of the progestin excessively blunted the anabolic impacts of endogenous androgens. The simple substitution of the anti-androgenic progestin with a neutral or an androgenic molecule mitigated the detrimental effect on bone turnover, re-establishing the balance between formation and absorption, like the phenomenon seen in the control women. Interestingly, the administration of a progestin-only contraceptive did not reduce bone turnover, and induced a positive balance between bone formation and reabsorption.

Aside from EE, there were no data on CHC containing E2 or E4. Neither estrogen molecule markedly stimulates SHBG. In addition, E2 binds mostly to circulating SHBG, which, being partly bound, leaves testosterone freer, and capable of acting on its receptors [66]. Data obtained in women above 21 years of age support this view.

#### 3.2.2. Bone Turnover in Women >21 Years of Age

In post-pubertal women, bone mass is considered stable, and bone neoformation matches bone reabsorption. This result also emerged from the analysis of bone markers of control women, confirming the previous knowledge [10].

CHC decreased bone turnover with a slight differential effect, depending on the estrogen and progestin molecules used. The balanced decline of bone formation and absorption suggests bone conservation and is unlikely to indicate a detrimental effect of CHC [55]. It was reported that long-term use of CHCs may harm BMD accumulation [64,67]. The possibility that the divergent results depend on the components of CHCs administered cannot be excluded [9,62,64,67,68,69]. The present analysis indicates that CHCs containing EE and E4 decrease bone turnover, with a similar effect seen on bone formation and absorption. The finding of similar reductions in both types of markers supports a net neutral impact on bone mass. Although limited, the data on CHC containing E2 appear to be different. During E2-CHC administration, absorption decreased more than formation, possibly leading to a positive effect on bone. Thus, E2-based CHC may emerge as being more appropriate in conditions where, for the most part, bone formation is needed, as for cases involving pubertal girls or the recovery from hypoestrogenic states.

As for conditions of reproductive health, the androgenic properties of progestin do not play a critical role. Effects observed were similar with neutral, androgenic, and anti-androgenic progestins. Indeed, with anti-androgenic progestins, absorption seemed more reduced than formation, but the effect was probably consequent to the estradiol-carrying effect, with most anti-androgenic progestins being combined in this manner. Interestingly, in the E4 group, the addition of an anti-androgenic progestin decreased bone formation, while the adjunct of an androgenic progestin did not affect it.

All of these results are reassuring and consistent with the literature data showing a neutral effect of CHC on bone mass. Furthermore, the capability of CHC to reduce bone turnover, even absent modifications in BMD, could be considered a positive result for women of reproductive age, with an elevated turnover representing a risk factor for bone fractures, independent of BMD values. Accordingly, CHCs may contribute to reducing the risk of fractures, particularly in those women associated with a low BMD.

### 3.3. Mechanistic Insights into the Distinct Effects of Estrogens and Progestins on Bone Turnover

Estrogens and progestins regulate bone turnover through distinct mechanisms. Estrogen primarily inhibits osteoclast-mediated resorption via the RANKL/RANK (receptor activator of nuclear factor-kB) and OPG (osteoprotegerin) axis, enhancing osteoblast survival by stimulating gene expression related to OPG synthesis in osteoblasts [48]. Progestins may indirectly affect bone turnover by modulating estrogen receptor activity or acting on progesterone receptors on osteoblasts. The inhibitory effects of CHCs on bone-formation markers, such as P1NP, are mediated by the downregulation of hepatic IGF-1, a mechanism that relies on estrogen metabolism [51]. Progestins seem to have a limited direct effect on hepatic IGF-1 production; however, their interactions with estrogens may enhance or diminish these effects based on the specific contraceptive formulation used [51]. The roles of growth hormone and estrogen in bone remodeling are synergistic, with insulin-like growth factor 1 serving as a central mediator [53]. The degree to which progestins affect this axis is not well established. Certain progestins exhibit androgenic activity, and androgens significantly influence women’s bone metabolism via both direct and indirect mechanisms. Androgens can directly interact with androgen receptors in bone cells. Osteoblasts, which are the cells involved in bone formation, exhibit the presence of androgen receptors (AR). Androgens binding to these receptors stimulate osteoblast proliferation and differentiation, resulting in enhanced bone formation [65,70]. Additionally, androgens can directly suppress osteoclastogenesis, the process by which osteoclasts, the cells responsible for bone tissue resorption, are formed. This inhibition reduces bone resorption, thus maintaining bone density [65,70,71]. Androgens indirectly influence the production of growth factors, including insulin-like growth factors (IGFs), which are essential for bone growth and remodeling. Androgens indirectly promote bone formation and maintenance by modulating IGF levels [72]. Future research should analyze these interactions, especially in formulations that combine low-dose estrogens with different progestins, to enhance therapeutic strategies that optimize efficacy while preserving skeletal integrity.

### 3.4. Strengths and Weaknesses

This meta-analysis combines a large amount of evidence from various randomized clinical trials, allowing for a robust evaluation of the impact of CHC on bone-turnover markers. This comprehensive synthesis enhances statistical power, provides precise estimations of treatment effects, and reduces the influence of individual study biases, thereby strengthening the reliability of conclusions. Examining diversity among trials provides valuable insights into the factors influencing bone-turnover markers. However, the inherent heterogeneity among the included studies poses a significant challenge to interpreting our findings. This analysis aimed to capture differences among various estrogen and progestin combinations; however, the diversity in study designs, populations, dosing regimens, follow-up periods, and publication bias hindered the ability to draw definitive conclusions. The studies encompassed randomized controlled trials and prospective cohort investigations, each exhibiting different levels of methodological rigor and susceptibility to bias. The variations in participant age, baseline bone health, and the specific hormonal formulations examined further complicated direct comparisons. Inconsistencies in the selection of bone metabolism markers added complexity to interpreting the magnitude and direction of the effects. Standardized mean change and meta-regression techniques have sought to address some of these issues, but residual confounding cannot be entirely ruled out. This meta-analysis may also be limited by the potential for publication bias, which could have affected the results. Certain markers, including P1NP, DPD, and CTX, demonstrated signs of bias. This finding may indicate a tendency to report outcomes that correspond with the anticipated effects of hormonal contraceptives on bone metabolism. Future research should address these limitations by ensuring the publication of negative studies and adopting open data-sharing practices to enhance transparency and minimize bias. Furthermore, there is a risk of population fallacy, which could hinder the direct relevance of aggregated findings to individual cases or specific clinical environments. Navigating these strengths and weaknesses is crucial to the accurate interpretation and application of the meta-analysis results. To interpret the data, we categorized the potency of estrogens based on their effect on SHBG. This type of analysis may have brought some distortion, given the relative inability of some estrogens, like E4, to act on liver enzymes. Because androgens exert an anabolic effect, we tried to categorize the types of progestins on the basis of their androgenic and anti-androgenic properties. This sub-division approximates the effect of androgens and does not consider the many other variables that characterize each single progestin molecule [73]. We selected the most-used markers of bone turnover, particularly those more representative of bone formation and absorption. Other less specific indices (ALP, urinary calcium) showed similar trends without reaching statistical significance. Most of the data are reported in supplemental figures and tables. Modifications of BMD identified in these studies, selected because they were relevant to the analyzed bone markers, were also collected and evaluated (Supplemental Figures S9 and S10). No apparent modification of BMD during CHC was observed. Nevertheless, this is not a systematic review and meta-analysis of BMD.

### 3.5. Relevance and Generalizability of the Findings

The results of this comprehensive analysis, which included a wide range of randomized clinical trials involving different populations, interventions, and settings, indicate a strong and widely applicable understanding of how CHCs affect bone turnover. Nevertheless, it is essential to be cautious when applying these findings universally, as individual variations, contextual factors, and evolving practices within specific demographics or clinical scenarios may influence the generalizability of these conclusions. In this review, for example, there were no studies performed in hypoestrogenic young women, women with weight disturbances, those suffering from polycystic ovary syndrome, or those in perimenopause.

### 3.6. Unanswered Questions and Future Research

Our research highlights the importance of different types of estrogens and progestin as to bone metabolism. It should be noted that there is insufficient long-term evidence in the existing literature to completely undercover the effects of the different estrogens and the different progestins on the bones of women of different reproductive ages. Based on current knowledge, CHCs affect bone metabolism by slowing the turnover process, which can benefit adults and older individuals. However, the reduction in bone turnover in girls in full pubertal development is still uncertain, with the marked decrease in bone formation possibly hampering an adequate peak bone-mass. It would be desirable to conduct prospective randomized studies with long-term follow-up to evaluate the impact of CHCs on bone health, while considering the characteristics of estrogens and progestins and the subjects’ different reproductive ages.

## 4. Materials and Methods

### 4.1. Study Design and Inclusion Criteria

This systematic review and meta-analysis examined the impact of hormonal contraception on bone turnover in patients of childbearing age. The analysis focused on markers of bone formation and reabsorption. The considered markers of bone formation were total and bone-specific alkaline phosphatase (ALP), along with the markers of new collagen formation osteocalcin (OC) and P1NP [74,75,76,77,78] (Supplemental Table S4). The markers of bone reabsorption considered were diet-independent indices of bone collagen degradation like pyridinoline (PYD) and the more bone-specific deoxypyridinoline (DPD), C-terminal telopeptide (CTX), N-terminal telopeptide (NTX), sclerostin (SOST), and tartrate-resistant acid phosphatase 5b (TRACP 5b) [74,75,79,80,81,82,83,84] (Supplemental Table S4). Data on urinary and serum calcium were also collected. In the included studies, serum calcium refers to total calcium. Data on bone mineral density variation (BMD) were retrieved as well. Searches, which were conducted in December 2024, focused on published randomized controlled trials (RCTs) and prospective studies. Eligibility criteria included specific data on bone-turnover marker dosages and CHC therapy in healthy, reproductive-age women under 45 years of age. In this study, “healthy” refers to women with menstrual cycles and no known hormonal or metabolic issues. Excluded were case reports; studies that were not human studies; non-prospective studies; review studies; studies not in English, German, French, or Spanish; and those outside our target population (healthy women under 45). The authors of the studies were not contacted to provide any additional data. This study required no ethical approval, as it involved only published and anonymized data. The protocol was registered in PROSPERO (CRD42023460827).

### 4.2. Data Source and Search Strategy

Adhering to PRISMA guidelines [85] and the PICO framework, the search included terms like OC, ALP, CTX, NTX, PYD, DPD, P1NP, sclerostin (SOST), tartrate-resistant acid phosphatase 5b (TRACP 5b), and oral contraception. Specific queries are listed in Supplemental Table S5. The databases, searched across a range from their inception to December 2024, included PubMed/Medline, Embase, Scopus, the Cochrane Database, the International Clinical Trials Registry, and ClinicalTrials.gov.

### 4.3. Selection of Relevant Studies and Quality Assessment

Two researchers (AT and APL) independently reviewed studies using a predefined template and utilizing Zotero for full-text management. When data overlap occurred, the more informative or higher-quality study was chosen. Alternatively, if both reports described distinct facets of the same study, they were retained in their entirety. Disagreements were resolved by a third reviewer (AC). Quality was assessed using the Cochrane tool for RCTs [86] and the Newcastle–Ottawa Scale for case-control and cohort studies [87].

### 4.4. Data Extraction

AT and APL independently extracted data using a predefined form. Data included general information; hormonal and non-hormonal contraceptive use; administration times; and dosages of bone-turnover markers like CTX, NTX, PYD, DPD, ALP, OC, Sclerostin, TRACP 5b, and P1NP. The androgenic effects of progestins were assessed by assigning anti-androgenic progestins a value of −1, neutral progestins 0, and pro-androgenic progestins 1. These values were multiplied by the progestin dose in micrograms and normalized with min–max normalization (Supplemental Table S6). The estrogenic effect on SHBG was evaluated by comparing the effect of a 1mg dose of estrogen to estradiol’s potency, using values established in the literature (Supplemental Table S7) [18,88]. The potency was then multiplied by the estrogen dosage in micrograms and normalized with min–max normalization.

### 4.5. Data Analysis

R (version 4.3.3) was used for analysis. Significance was set at a *p*-value of <0.05. Standardized mean changes (SMC) were calculated to assess the effect sizes of bone-turnover markers before and after hormonal contraceptive treatment [89,90]. The SMC serves as an effective instrument for synthesizing data from pre-test–post-test designs, facilitating direct comparisons across studies employing diverse methodologies, including those lacking control groups [90]. Standardizing effect sizes through SMC enables meaningful comparisons across diverse studies and addresses baseline-level variations. This approach translates treatment effects into a universally comprehensible metric, facilitating interpretation and allowing for comparisons of effect sizes across various treatment arms from different studies. Publication bias was assessed with funnel plots and statistical tests [91,92,93]. Heterogeneity was checked using the I-square index and Cochran Q tests [94], with a random or fixed effects model applied based on heterogeneity. Outcomes were described using SMCs and 95% CIs, and sensitivity analyses evaluated the reliability of combined effects. We conducted a sensitivity analysis that stratified studies by type of ALP and age and performed a leave-one-out analysis within strata containing more than three studies to ensure the robustness and consistency of our results. Differences among the strata in the forest plots were assessed by a mixed-effects meta-regression model that evaluated the contributions of moderators to the variability in effect sizes. The significance of the moderators was assessed using the omnibus test of moderators (QM statistic). Both univariate and multivariate meta-regression analyses were conducted to explore potential sources of heterogeneity and assess the influence of study-level covariates on the outcomes. The meta-regressions aimed to examine the relationships between the SMCs of bone-turnover markers and explanatory variables, including the androgenic effects of progestins and the estrogenic effect on SHBG, and the publication, follow-up length, and maximum age. Variables were selected as potential sources of heterogeneity based on the prior literature [5,6,7,8,9].

## 5. Conclusions

In summary, the results emphasize the influence of CHC on bone turnover, suggesting a tendency towards reduced bone turnover. Differential effects on bone formation and absorption are essential. In this regard, CHCs containing natural estrogens and progestins with androgenic properties appear to be more advantageous than those containing EE and anti-androgenic progestins. These results may have important clinical implications for the prescription of CHC in the different reproductive phases of a woman’s life. The analysis leaves open the possibility of future studies dedicated to clarifying the exact effect of CHC on bone. The objective is to personalize CHC prescriptions, given considerations of bone health.

## Figures and Tables

**Table 1:** Key features of the included studies.

Study	Condition	Country	Study Period	Allocation	Age Range (Min–Max)
Orsolini 2023 [44]	Healthy	Brazil	2014–2020	Unknown	12–20
Caldeirão 2022 [13]	Healthy	Brazil	2014–2020	Unknown	15–20
Almstedt 2020 [14]	Healthy	USA	2016–2017	Systematic allocation	18–20
Tiedeken 2019 [15]	Healthy	USA	2012–2013	Random allocation	18–39
Rizzo 2019 [16]	Healthy	Brazil	2011–2017	Systematic allocation	12–20
Iltemir Duvan 2017 [17]	Breast feeding	Turkey	2009–2011	Systematic allocation	24–38
Mawet 2015 [18]	Healthy	Netherlands	2009	Random allocation	18–35
Hernandez-Juarez 2014 [43]	Healthy	Mexico	2011–2013	Systematic allocation	18–35
Di Carlo 2013 [19]	Healthy	Italy	2011	Single arm	21–34
Cibula 2012 [34]	Healthy	Czech Republic	2006–2008	Random allocation	15–20
Misra 2011 [46]	Healthy/amenorrhea	Multinational	2009–2010	Systematic and random allocation	12–18
Massaro 2010 [20]	Healthy	Italy	2008	Random allocation	23–34
Harel 2010 [21]	Healthy	USA	2008–2009	Systematic allocation	12–21
Lattakova 2009 [22]	Healthy	Slovakia	2007–2008	Systematic allocation	16–19
Gargano 2008 [23]	Healthy	Italy	2006	Random allocation	21–34
Kaunitz 2006 [35]	Healthy	USA	1997–2004	Unknown	25–35
Nappi 2005 [24]	Healthy	Italy	2002–2003	Random allocation	22–34
Paoletti 2004 [26]	Healthy	Italy	2003	Systematic allocation	20–30
Endrikat 2004 [27]	Healthy	Germany	1997–2001	Random allocation	20–38
Rome 2004 [25]	Healthy	USA	2002–2003	Systematic allocation	12–18
Nappi 2003 [28]	Healthy	Italy	2000–2002	Random allocation	22–34
Paoletti 2000 [29]	Healthy	Italy	1997–1999	Systematic and random allocation	22–30
Naessen 1995 [30]	Healthy	Sweden	1993–1994	Random allocation	20–45
Mais 1993 [31]	Healthy	Italy	1991–1992	Single arm	20–30
Etzrodt 1990 [39]	Healthy	Germany	1987–1988	Unknown	18–35
Hurley 1989 [38]	Healthy	USA	1987–1988	Systematic allocation	21–29
Sadik 1985 [33]	Healthy	Multinational	1983–1984	Systematic and random allocation	18–39
Ahrén 1981 [36]	Healthy	Sweden	1979–1980	Unknown	18–35
Bamji 1981 [42]	Healthy	India	1978–1979	Single arm	Unknown
Roy 1980 [32]	Healthy	USA	1976–1978	Systematic and random allocation	17–33
Amatayakul 1980 [37]	Healthy	Thailand	1976–1977	Single arm	18–29
Amatayakul 1978 [45]	Healthy	Thailand	1976–1977	Single arm	18–29
Brügmann 1975 [41]	Healthy	Germany	1973–1974	Single arm	17–38
García 1968 [40]	Healthy	USA	1966–1968	Single arm	Unknown

**Table 2:** Pooled standardized mean changes (SMCs) in bone markers (formation and resorption) among healthy women before and after hormonal contraceptive treatment, stratified by estrogen and progestin type and participant maximum age of enrollment (≤21 vs. >21 years).

Variable	SMC (CI.95)/*p*-Value (all)	SMC (CI.95)/*p*-Value (≤21)	SMC (CI.95)/*p*-Value (>21)
Estrogens			
Bone-formation markers			
OC	<0.001 (*)	0.944 (*)	<0.001 (*)
E2V	−0.10 (−0.47/0.27)	---	−0.10 (−0.47/0.27)
E4	−0.43 (−0.76/−0.10)	---	−0.43 (−0.76/−0.10)
EE	−0.54 (−0.64/−0.43)	−0.63 (−0.77/−0.49)	−0.42 (−0.61/−0.24)
Only progestin	0.55 (0.07/1.04)	---	0.55 (0.07/1.04)
Controls	−0.12 (−0.35/0.10)	−0.65 (−1.06/−0.23)	0.01 (−0.20/0.21)
ALP (bone)	0.501 (*)	0.321 (*)	0.949 (*)
EE	−0.39 (−0.67/−0.11)	−0.55 (−0.86/−0.24)	−0.06 (−0.47/0.35)
Only progestin	0.13 (−0.14/0.40)	0.13 (−0.14/0.40)	---
Controls	−0.40 (−0.93/0.14)	−0.56 (−1.27/0.16)	−0.03 (−0.42/0.35)
ALP (all)	0.134 (*)	0.321 (*)	0.208 (*)
E2	−0.41 (−0.78/−0.04)	---	−0.41 (−0.78/−0.04)
E2EN	−0.23 (−0.63/0.16)	---	−0.23 (−0.63/0.16)
EE	−0.46 (−0.62/−0.30)	−0.55 (−0.86/−0.24)	−0.42 (−0.60/−0.24)
EEME	−0.61 (−1.47/0.25)	---	−0.61 (−1.47/0.25)
EES	0.00 (−0.46/0.46)	---	0.00 (−0.46/0.46)
Only progestin	0.02 (−0.23/0.27)	0.13 (−0.14/0.40)	−0.02 (−0.33/0.30)
Controls	−0.29 (−0.70/0.11)	−0.56 (−1.27/0.16)	−0.05 (−0.19/0.09)
PINP	0.001 (*)	0.083 (*)	---
E2	0.12 (−0.10/0.33)	---	0.12 (−0.10/0.33)
EE	−0.55 (−0.83/−0.26)	−0.55 (−0.83/−0.26)	---
Controls	−0.20 (−0.50/0.10)	−0.20 (−0.50/0.10)	---
Bone-resorption markers			
DPD	<0.001 (*)	0.436 (*)	<0.001 (*)
E2V	−1.57 (−2.13/−1.02)	---	−1.57 (−2.13/−1.02)
EE	−0.33 (−0.43/−0.22)	−0.07 (−0.22/0.08)	−0.58 (−0.73/−0.42)
Only progestin	−0.07 (−0.34/0.20)	−0.07 (−0.34/0.20)	---
Controls	−0.02 (−0.14/0.10)	0.07 (−0.09/0.23)	−0.13 (−0.31/0.05)
PYD	0.003 (*)	---	0.003 (*)
E2V	−0.69 (−1.10/−0.27)	---	−0.69 (−1.10/−0.27)
EE	−0.37 (−0.53/−0.21)	---	−0.37 (−0.53/−0.21)
Controls	−0.05 (−0.23/0.13)	---	−0.05 (−0.23/0.13)
NTX	0.084 (*)	0.149 (*)	---
EE	−0.43 (−0.74/−0.12)	−0.45 (−1.37/0.47)	−0.43 (−0.76/−0.10)
Controls	−1.65 (−3.00/−0.30)	−1.65 (−3.00/−0.30)	---
CTX	0.02 (*)	0.908 (*)	<0.001 (*)
E2	0.25 (0.02/0.48)	---	0.25 (0.02/0.48)
E4	−0.41 (−0.63/−0.20)	---	−0.41 (−0.63/−0.20)
EE	−0.38 (−0.69/−0.07)	−0.26 (−0.48/−0.03)	−1.33 (−1.93/−0.73)
Controls	−0.23 (−0.56/0.10)	−0.23 (−0.56/0.10)	---
Progestins			
Bone-formation markers			
OC	0.005 (*)	0.628 (*)	0.004 (*)
Controls	−0.12 (−0.35/0.10)	−0.65 (−1.06/−0.23)	0.01 (−0.20/0.21)
Anti-androgenic	−0.61 (−0.79/−0.43)	−0.69 (−0.87/−0.50)	−0.59 (−0.88/−0.30)
Neutral	−0.23 (−1.10/0.65)	−1.11 (−2.22/0.01)	0.07 (−0.82/0.97)
Pro-androgenic	−0.31 (−0.47/−0.15)	−0.54 (−0.75/−0.33)	−0.18 (−0.36/−0.01)
ALP (bone)	0.059 (*)	0.085 (*)	0.084 (*)
Controls	−0.40 (−0.93/0.14)	−0.56 (−1.27/0.16)	−0.03 (−0.42/0.35)
Anti-androgenic	−0.79 (−1.15/−0.44)	−0.97 (−1.20/−0.73)	−0.42 (−0.81/−0.04)
Neutral	0.02 (−0.43/0.47)	0.02 (−0.43/0.47)	
Pro-androgenic	−0.16 (−0.37/0.05)	−0.27 (−0.48/−0.06)	0.14 (−0.18/0.46)
ALP (all)	0.087 (*)	0.085 (*)	0.360 (*)
Controls	−0.29 (−0.70/0.11)	−0.56 (−1.27/0.16)	−0.05 (−0.19/0.09)
Anti-androgenic	−0.64 (−1.03/−0.26)	−0.97 (−1.20/−0.73)	−0.31 (−0.57/−0.04)
Neutral	−0.02 (−0.23/0.20)	0.02 (−0.43/0.47)	−0.12 (−0.41/0.17)
Pro-androgenic	−0.35 (−0.49/−0.21)	−0.27 (−0.48/−0.06)	−0.36 (−0.54/−0.19)
PINP	0.001 (*)	0.083 (*)	---
Controls	−0.20 (−0.50/0.10)	−0.20 (−0.50/0.10)	---
Neutral	0.12 (−0.10/0.33)		0.12 (−0.10/0.33)
Pro-androgenic	−0.55 (−0.83/−0.26)	−0.55 (−0.83/−0.26)	---
Bone-resorption markers			
DPD	<0.001 (*)	0.436 (*)	<0.001 (*)
Controls	−0.02 (−0.14/0.10)	0.07 (−0.09/0.23)	−0.13 (−0.31/0.05)
Anti-androgenic	−0.84 (−1.05/−0.63)	---	−0.84 (−1.05/−0.63)
Neutral	−0.21 (−0.46/0.03)	−0.07 (−0.34/0.20)	−0.88 (−1.46/−0.30)
Pro-androgenic	−0.19 (−0.31/−0.06)	−0.07 (−0.22/0.08)	−0.41 (−0.63/−0.20)
PYD	<0.001 (*)	---	<0.001 (*)
Controls	−0.05 (−0.23/0.13)	---	−0.05 (−0.23/0.13)
Anti-androgenic	−0.56 (−0.75/−0.37)	---	−0.56 (−0.75/−0.37)
Neutral	−0.63 (−1.17/−0.10)	---	−0.63 (−1.17/−0.10)
Pro-androgenic	−0.19 (−0.40/0.02)	---	−0.19 (−0.40/0.02)
NTX	0.225 (*)	0.149 (*)	---
Controls	−1.65 (−3.00/−0.30)	−1.65 (−3.00/−0.30)	---
Neutral	−0.45 (−1.37/0.47)	−0.45 (−1.37/0.47)	---
Pro-androgenic	−0.43 (−0.76/−0.10)		−0.43 (−0.76/−0.10)
CTX	0.020 (*)	0.978 (*)	0.001 (*)
Controls	−0.23 (−0.56/0.10)	−0.23 (−0.56/0.10)	---
Anti-androgenic	−0.47 (−0.84/−0.10)	−0.22 (−0.50/0.06)	−0.68 (−1.27/−0.10)
Neutral	0.25 (0.02/0.48)	---	0.25 (0.02/0.48)
Pro-androgenic	−0.33 (−0.57/−0.09)	−0.28 (−0.61/0.06)	−0.43 (−0.78/−0.09)

The *p*-values indicate differences among strata (QM statistic) (*). Please refer to the Supplementary Materials for detailed information on the single-arm data.

**Table 3:** Pooled standardized mean changes (SMCs) in all bone-formation and bone-resorption markers among healthy women before and after hormonal contraceptive treatment, stratified by estrogen and progestin type and the maximum age of enrolled women (≤21 vs. >21 years). The *p*-values indicate differences among strata (QM statistic) (*).

Variable	SMC (CI.95)/*p*-Value (all)	SMC (CI.95)/*p*-Value (≤21)	SMC (CI.95)/*p*-Value (>21)
Estrogens			
Bone-formation markers (all)	<0.001 (*)	0.027 (*)	0.001 (*)
Controls	−0.17 (−0.33/−0.02)	−0.43 (−0.75/−0.11)	−0.03 (−0.15/0.08)
E2	−0.04 (−0.28/0.20)	---	−0.04 (−0.28/0.20)
E2V	−0.10 (−0.47/0.27)	---	−0.10 (−0.47/0.27)
E2EN	−0.23 (−0.63/0.16)	---	−0.23 (−0.63/0.16)
E4	−0.43 (−0.76/−0.10)	---	−0.43 (−0.76/−0.10)
EE	−0.49 (−0.59/−0.39)	−0.58 (−0.72/−0.44)	−0.42 (−0.54/−0.29)
EEME	−0.61 (−1.47/0.25)	---	−0.61 (−1.47/0.25)
EES	0.00 (−0.46/0.46)	---	0.00 (−0.46/0.46)
Only progestin	0.11 (−0.13/0.36)	0.13 (−0.14/0.40)	0.12 (−0.18/0.42)
Bone-resorption markers (all)	<0.001 (*)	0.833 (*)	<0.001 (*)
Controls	−0.07 (−0.16/0.02)	−0.21 (−0.52/0.10)	−0.09 (−0.22/0.04)
E2	0.25 (0.03/0.47)	---	0.25 (0.02/0.48)
E2V	−1.00 (−1.33/−0.67)	---	−1.11 (−1.98/−0.24)
E4	−0.41 (−0.63/−0.20)	---	−0.41 (−0.63/−0.20)
EE	−0.33 (−0.40/−0.26)	−0.22 (−0.40/−0.04)	−0.49 (−0.62/−0.37)
Only progestin	−0.07 (−0.34/0.20)	−0.07 (−0.34/0.20)	---
Progestins			
Bone-formation markers (all)	<0.001 (*)	0.010 (*)	<0.001 (*)
Controls	−0.17 (−0.33/−0.02)	−0.43 (−0.75/−0.11)	−0.03 (−0.15/0.08)
Anti-androgenic	−0.63 (−0.79/−0.46)	−0.80 (−0.94/−0.65)	−0.51 (−0.74/−0.29)
Neutral	0.02 (−0.11/0.16)	−0.31 (−1.02/0.40)	0.02 (−0.14/0.18)
Pro-androgenic	−0.34 (−0.44/−0.25)	−0.42 (−0.58/−0.27)	−0.31 (−0.44/−0.18)
Bone-resorption markers (all)	<0.001 (*)	0.993 (*)	<0.001 (*)
Controls	−0.07 (−0.16/0.02)	−0.21 (−0.52/0.10)	−0.09 (−0.22/0.04)
Anti-androgenic	−0.54 (−0.65/−0.44)	−0.22 (−0.50/0.06)	−0.70 (−0.89/−0.51)
Neutral	−0.03 (−0.18/0.13)	−0.10 (−0.36/0.16)	−0.12 (−0.61/0.37)
Pro-androgenic	−0.24 (−0.32/−0.15)	−0.22 (−0.48/0.04)	−0.34 (−0.47/−0.22)

## Data Availability

All data were extracted from previously published studies; thus, they are publicly available. Moreover, this article and the Supplementary Materials include all the data used.

## Supplementary Materials

The following supporting information can be downloaded at: https://www.mdpi.com/article/10.3390/ph18010061/s1. Figure S1: Details of research quality assessments; Figure S2: Forest plots of osteocalcin, alkaline phosphatase, and P1NP among healthy women; Figure S3: Forest plots of alkaline phosphatase levels, encompassing nonspecific and inclusive categories (nonspecific and bone-specific) among healthy women; Figure S4: Forest plots of CTX, NTX, DPD, and PYD in healthy women; Figure S5: Univariate meta-regressions that examine the androgenic effects of progestins when combined with estrogens in treating healthy women; Figure S6: Univariate meta-regressions that examine the estrogenic effect on sex hormone-binding globulin (SHBG), when combined with estrogens, on bone mineral density (BMD) in treating healthy women; Figure S7: Multivariate meta-regressions (adjustment for estrogens, publication, follow-up length, and maximum age) that examine the androgenic effects of progestins when combined with estrogens in treating healthy women; Figure S8: Forest plots of urine and serum calcium in a cohort of healthy women; Figure S9: Forest plots representing the bone-mass density (BMD) assessment in different anatomical locations of healthy women among the included studies; Figure S10: Univariate meta-regressions of bone-mass density (BMD) as the dependent variable; Figure S11: Funnel plots and results of Egger’s test; Table S1: Study objectives; Table S2: Included and excluded studies, with reasons [7,8,13,14,15,16,17,18,19,20,21,22,23,24,25,26,27,28,29,30,31,32,33,34,35,36,37,38,39,40,41,42,43,44,45,46,52,65,95,96,97,98,99,100,101,102,103,104,105,106,107,108,109,110,111,112,113,114,115,116,117,118,119,120,121,122,123,124,125,126,127,128,129,130,131,132,133,134,135,136,137,138,139,140,141,142,143,144,145,146,147,148,149,150,151,152,153,154,155,156,157,158,159,160,161,162,163,164,165,166,167,168,169,170,171,172,173]; Table S3: Characteristics of the arms of the included studies; Table S4: List of biochemical markers included in the study [74,75,76,79,80]; Table S5: Overview of the database queries; Table S6: The androgenic effects of progestogens [174,175,176,177,178,179,180,181]; Table S7: Relative potency of estrogens [18,88].

## Abbreviations

ALP: Alkaline phosphatase; BMD: Bone mineral density; CEBM: Centre for Evidence-Based Medicine; CHC: Combined hormonal contraceptive; CI.95: 95% confidence interval; CMA: Chlormadinone acetate; CPA: Cyproterone acetate; CTX: C-terminal telopeptide; DHPA: Dihydroxyprogesterone acetophenide; DNG: Dienogest; DPD: Deoxypyridinoline; DRSP: Drospirenone; DSG: Desogestrel; E2-EN: Estradiol enanthate; E2: Estradiol; E2V: Estradiol valerate; E4: Estetrol; EE: Ethinylestradiol; EEME: Ethinylestradiol 3-methyl ether; EES: Ethinylestradiol sulfonate; ENG: Etonogestrel; GSD: Gestodene; LNG: Levonorgestrol; MPA: Medroxyprogesterone acetate; NET: Norethisterone; NGM: Norgestimate; NGMN: Norelgestromin; NTX: N-terminal telopeptide; OC: Osteocalcin; P1NP: N-terminal propeptide of procollagen type I; P4: Progesterone; PCOS: Polycystic Ovary Syndrome; PICO: Patients, Intervention, Comparator, and Outcome; PRISMA: Preferred Reporting Items for Systematic Reviews and Meta-Analyses; PYD: Pyridinoline; RCT: Randomized controlled trial; SGA: Segesterone acetate; SHBG: Sex hormone-binding globulin; SMC: Standardized mean change; SOST: Sclerostin; TRACP 5b: Tartrate-resistant acid phosphatase 5b.
