# Supplementary material for: Hormonal Contraception and Bone Metabolism: Emerging Evidence from a Systematic Review and Meta-Analysis of Studies on Post-Pubertal and Reproductive-Age Women
Source: Pharmaceuticals (Basel). 2025 Jan 8;18(1):61. doi: 10.3390/ph18010061 (PMC11768253; doi:10.3390/ph18010061)
Supplement: Supplementary file 1 [file pharmaceuticals-18-00061-s001.zip › pharmaceuticals-3309991-supplementary.pdf]

Supplemental material

Hormonal Contraception and Bone Metabolism: Emerging Evidence from a Systematic Review and Meta-Analysis of Studies on Post-Pubertal and Reproductive-Age Women

Alice Tassi (1), Ambrogio P Londero (2,3), Anjeza Xholli (4), Giulia Lanzolla (5,6), Serena Bertozzi (7), Luca Savelli (1,8), Federico Prefumo (3), Angelo Cagnacci (2,4)

Department affiliations:
1- Obstetrics and Gynecology Unit, Morgagni-Pierantoni Hospital, 47121 Forlì (FC), Italy;
2- Department of Neuroscience, Rehabilitation, Ophthalmology, Genetics, Maternal and Infant Health, University of Genoa, 16132 Genova (GE), Italy;
3- Obstetrics and Gynecology Unit, IRCCS Istituto Giannina Gaslini, 16147 Genova (GE), Italy;
4- Academic Unit of Obstetrics and Gynecology, IRCCS Ospedale San Martino, 16132 Genoa (GE), Italy;
5- Department of Clinical and Experimental Medicine, Endocrinology Unit II, University of Pisa and University Hospital of Pisa, 56126 Pisa (PI), Italy.;
6- Department of Orthopaedic Surgery, University of Pennsylvania, Perelman School of Medicine, Philadelphia 19104, PA, USA;
7- Breast Unit, University Hospital of Udine, 33100 Udine (UD), Italy; 8- Dipartimento di Scienze Mediche e Chirurgiche (DIMEC), University of Bologna, Bologna, Italy.
8- Dipartimento di Scienze Mediche e Chirurgiche (DIMEC), University of Bologna, 40138 Bologna, Italy

Corresponding author (also for reprint requests):
Dr Ambrogio P Londero (MD, PhD);
Department of Neuroscience, Rehabilitation, Ophthalmology, Genetics, Maternal and Infant Health, University of Genoa; Largo Rosanna Benzi, 10, 16132 Genova (GE), Italy;
and
Obstetrics and Gynecology Unit, IRCCS Istituto Giannina Gaslini, Via Gerolamo Gaslini, 5, 16147 Genova (GE), Italy;
E-mail: ambrogio.londero@gmail.com or ambrogiopietro.londero@unige.it

Figure S1- The details of research quality assessments are depicted in this graphic. Panel A) Uses the Cochrane Collaboration's tool for assessing the risk of bias to display information on the quality of randomized controlled trials. Panel B) This panel delves into the Newcastle-Ottawa Quality Assessment Scale for observational research. Unassigned points are displayed as blank white bars, while assigned points are depicted in filled grayscale bars.

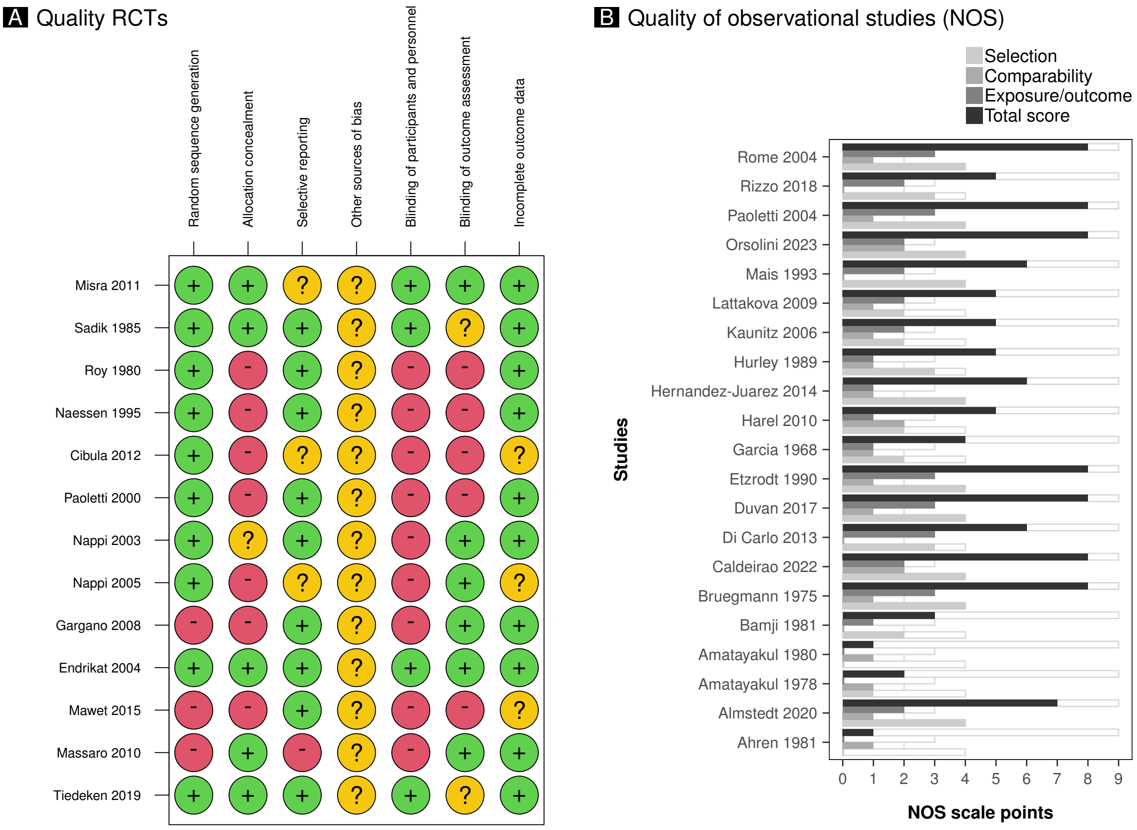

Figure S2- The visual representation depicts the forest plots of osteocalcin, alkaline phosphatase, and P1NP among a cohort of healthy women. Panel A shows osteocalcin standardized mean change (SMC) in healthy women before and after hormonal contraceptive treatment. Panel B shows bone-specific alkaline phosphatase SMC in healthy women before and after hormonal contraceptive treatment. Panel H shows the P1NP SMC in healthy women before and after hormonal contraceptive treatment. (*) Maximum age of women enrolled $\leq$ 21.

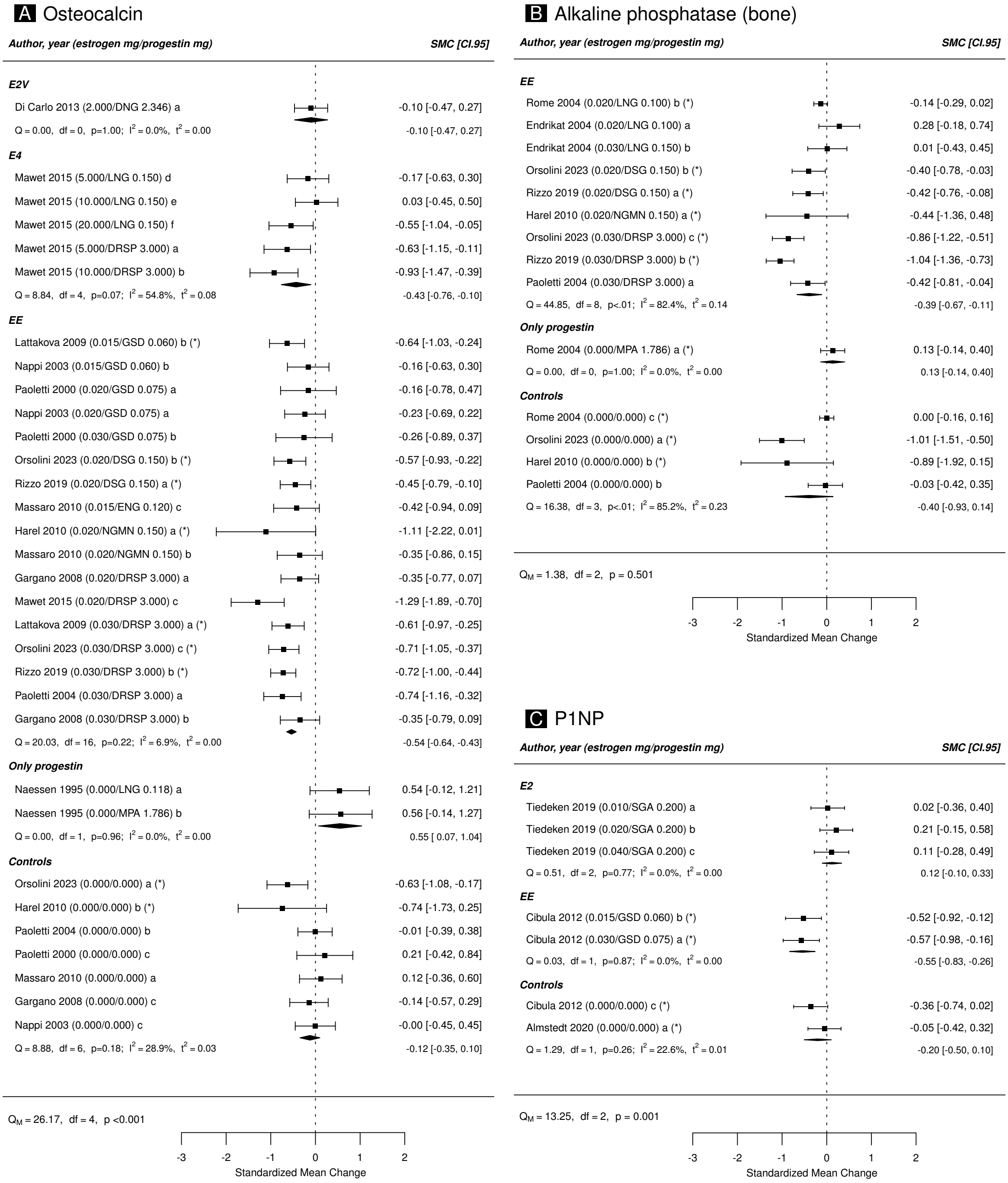

Figure S3- The visual representation illustrates the forest plots of alkaline phosphatase levels, encompassing nonspecific and inclusive categories (nonspecific and bone-specific) among healthy women. Panel A presents the standardized mean change (SMC) in nonspecific alkaline phosphatase levels among a cohort of healthy women before and after undergoing hormonal contraceptive therapy. Panel B presents the SMC of all alkaline phosphatases (containing nonspecific and bone-specific variants) in healthy women before and after hormonal contraceptive therapy. (*) Maximum age of women enrolled ≤ 21.

A Alkaline phosphatase (unspecified)

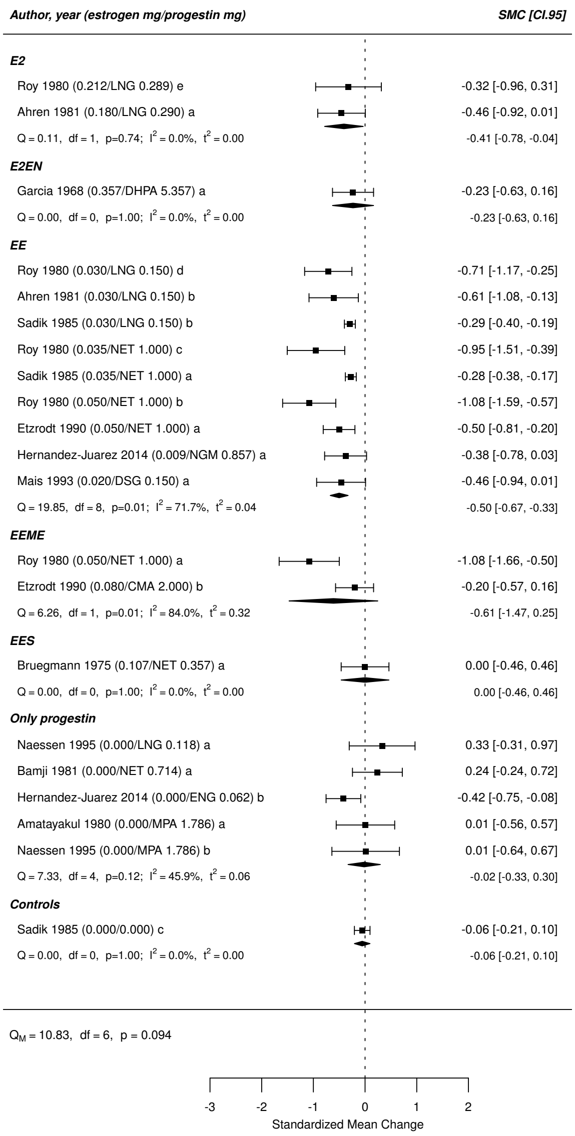

B Alkaline phosphatase (all)

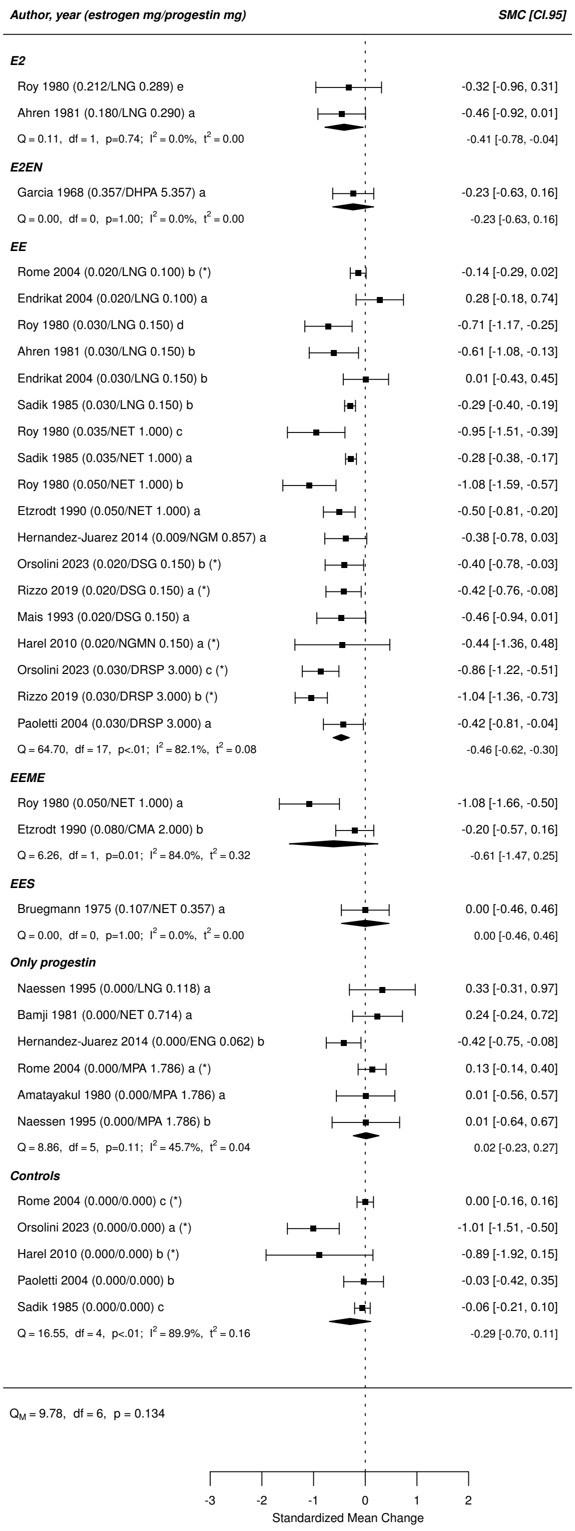

Figure S4- The illustration displays the forest plots of CTX, NTX, DPD, and PYD in a cohort of healthy women. Panel A depicts the CTX standardized mean change (SMC) in healthy women before and after hormonal contraceptive treatment. Panel B illustrates the NTX SMC before and after hormonal contraception in healthy women. Panel C represents PYD SMC in healthy women before and after hormonal contraceptive treatment. Panel D represents PYD SMC in healthy women before and after hormonal contraceptive treatment. (*) Maximum age of women enrolled $\leq$ 21. (*) Maximum age of women enrolled $\leq$ 21.

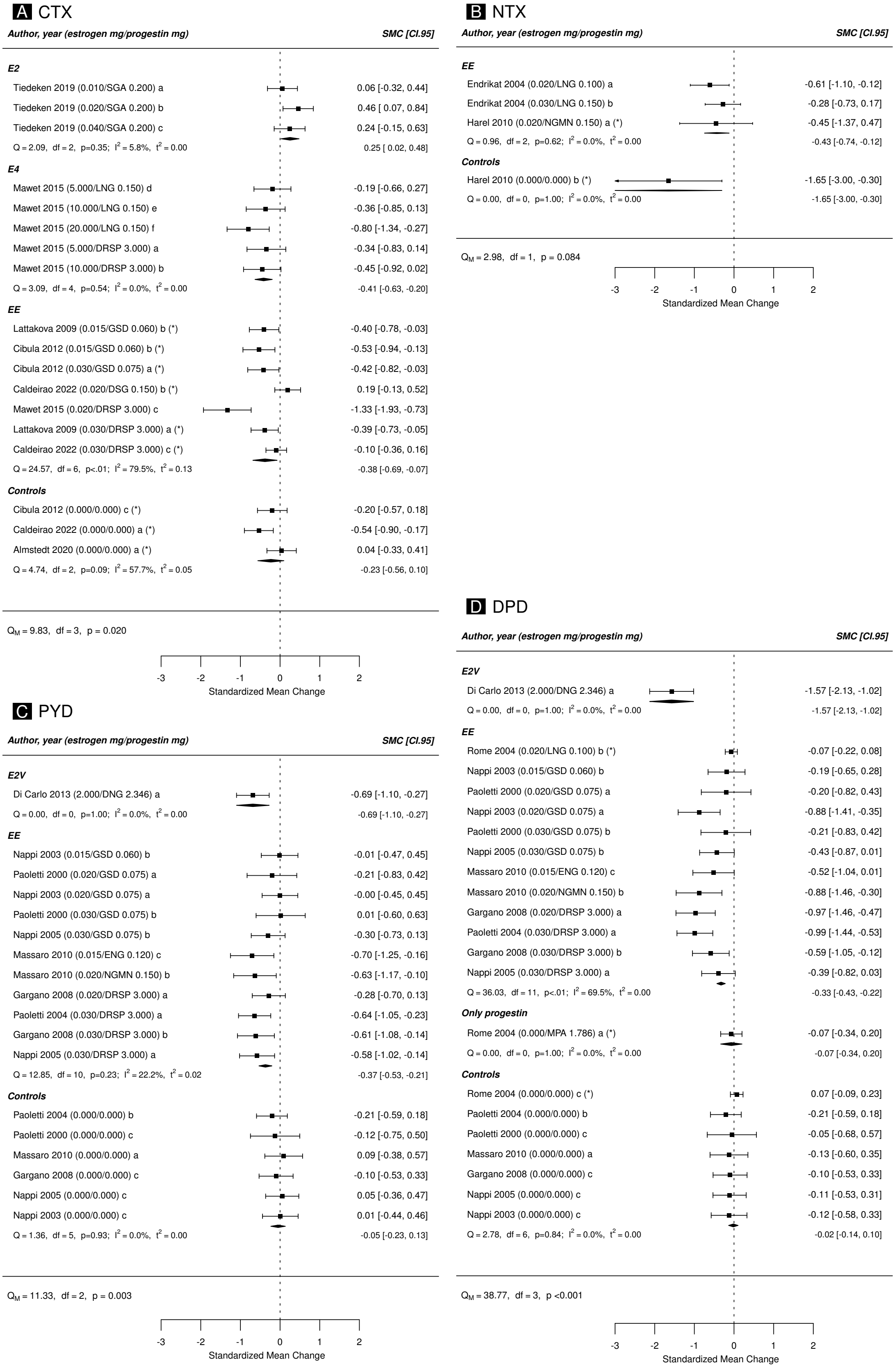

Figure S5- The figures illustrate the results of univariate meta-regressions that examine the androgenic effects of progestins when combined with estrogens in treating healthy women. The evaluation of the androgenic influence was carried out using the following methodology: progestins exhibiting anti-androgenic properties were assigned a numerical value of -1, progestins lacking androgenic effects were assigned a numerical value of 0, and progestins demonstrating pro-androgenic effects were assigned a numerical value of 1. The previously described androgenic effect (-1, 0, or 1) was multiplied by progestin dose values in micrograms, and the resulting value was then subjected to min-max normalization. Panel A shows the correlation between the androgenic effect of progestins and the standardized mean change of alkaline phosphatase (unspecified and bone-specific). Panel B shows the correlation between the androgenic effect of progestins and the standardized mean change of CTX.

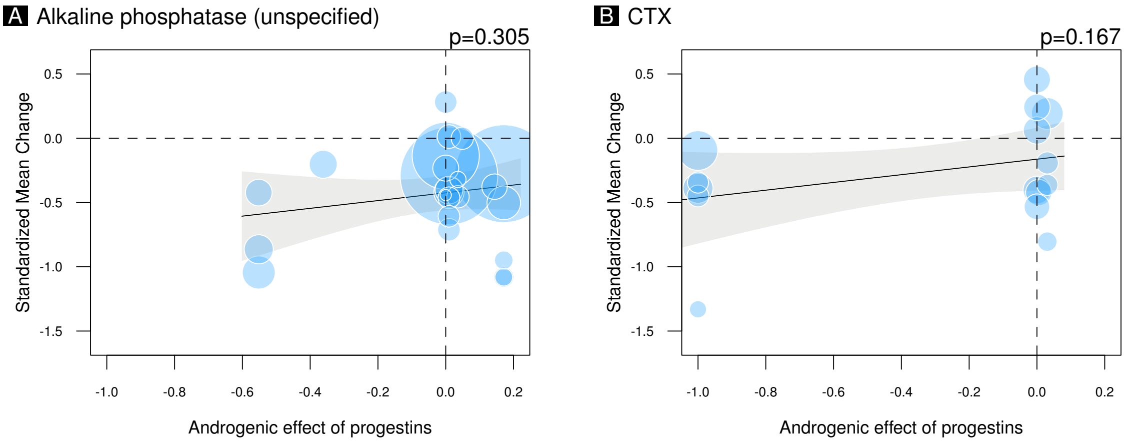

Figure S6- The figures illustrate the results of univariate meta-regressions that examine the estrogenic effect on sex hormone-binding globulin (SHBG) when combined with estrogens on bone mineral density (BMD) in treating healthy women. Panel A shows the correlation between the estrogenic effect on SHBG and the standardized mean change (SMC) of osteocalcin. Panel B shows the correlation between the estrogenic effect on SHBG and the SMC of alkaline phosphatase. Panel C shows the correlation between the estrogenic effect on SHBG and the SMC of P1NP. Panel D shows the correlation between the estrogenic effect on SHBG and the SMC of CTX. Panel E shows the correlation between the estrogenic effect on SHBG and the SMC of DPD. Panel F shows the correlation between the estrogenic effect on SHBG and the SMC of PYD.

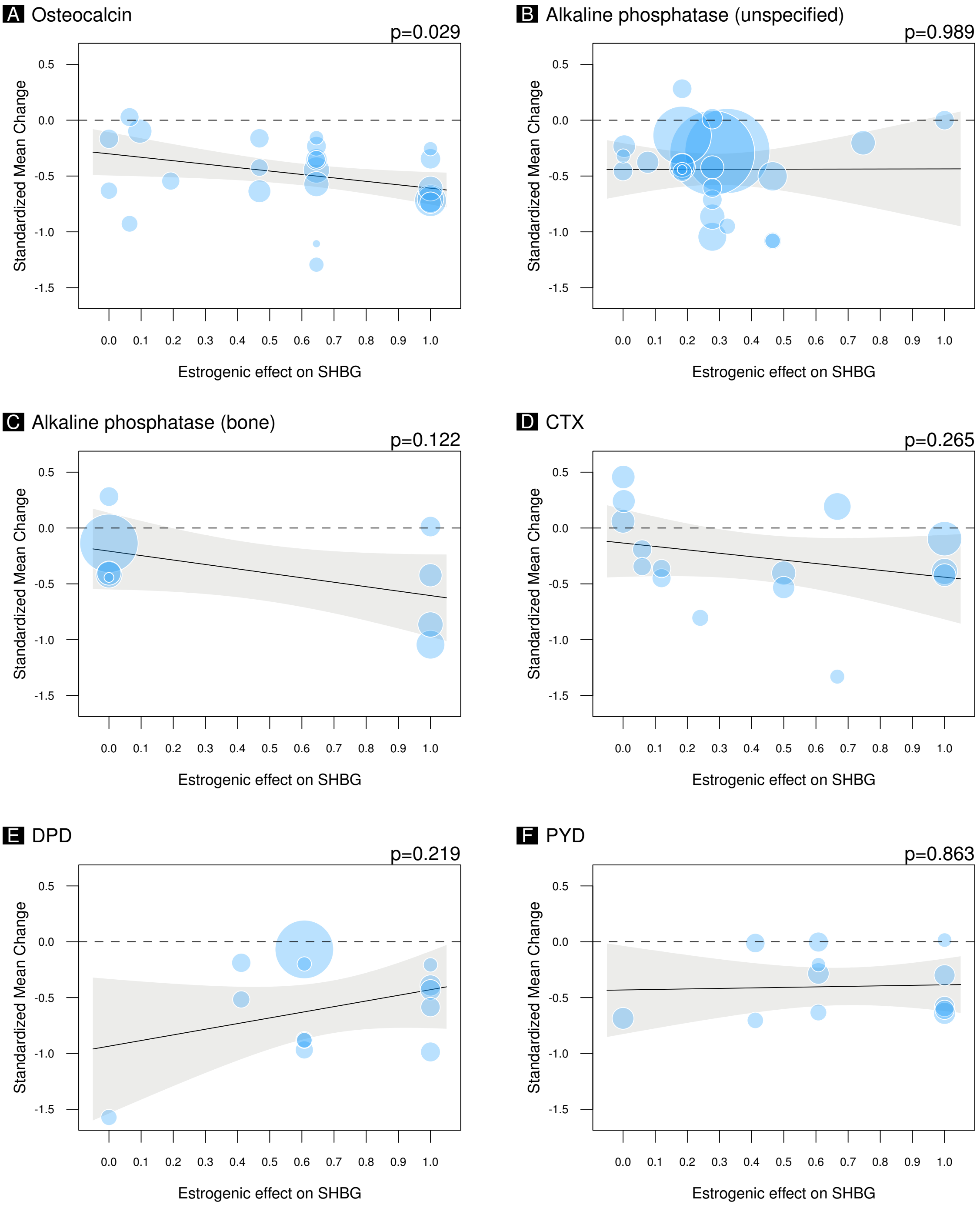

Figure S7- The figures illustrate the results of multivariate meta-regressions (adjustment for estrogens, publication, follow-up length, and maximum women age) that examine the androgenic effects of progestins when combined with estrogens in treating healthy women. The evaluation of the androgenic influence was carried out using the following methodology: progestins exhibiting anti-androgenic properties were assigned a numerical value of -1, progestins lacking androgenic effects were assigned a numerical value of 0, and progestins demonstrating pro-androgenic effects were assigned a numerical value of 1. The previously described androgenic effect (-1, 0, or 1) was multiplied by progestin dose values in micrograms, and the resulting value was then subjected to min-max normalization. Panel A shows the correlation between the androgenic effect of progestins and the standardized mean change of osteocalcin. Panel B shows the correlation between the androgenic effect of progestins and the standardized mean change of alkaline phosphatase (unspecified and bone-specific). Panel C shows the correlation between the androgenic effect of progestins and the standardized mean change of alkaline phosphatase (only bone-specific). Panel D shows the correlation between the androgenic effect of progestins and the standardized mean change of CTX. Panel E shows the correlation between the androgenic effect of progestins and the standardized mean change of DPD. Panel F shows the correlation between the androgenic effect of progestins and the standardized mean change of PYD.

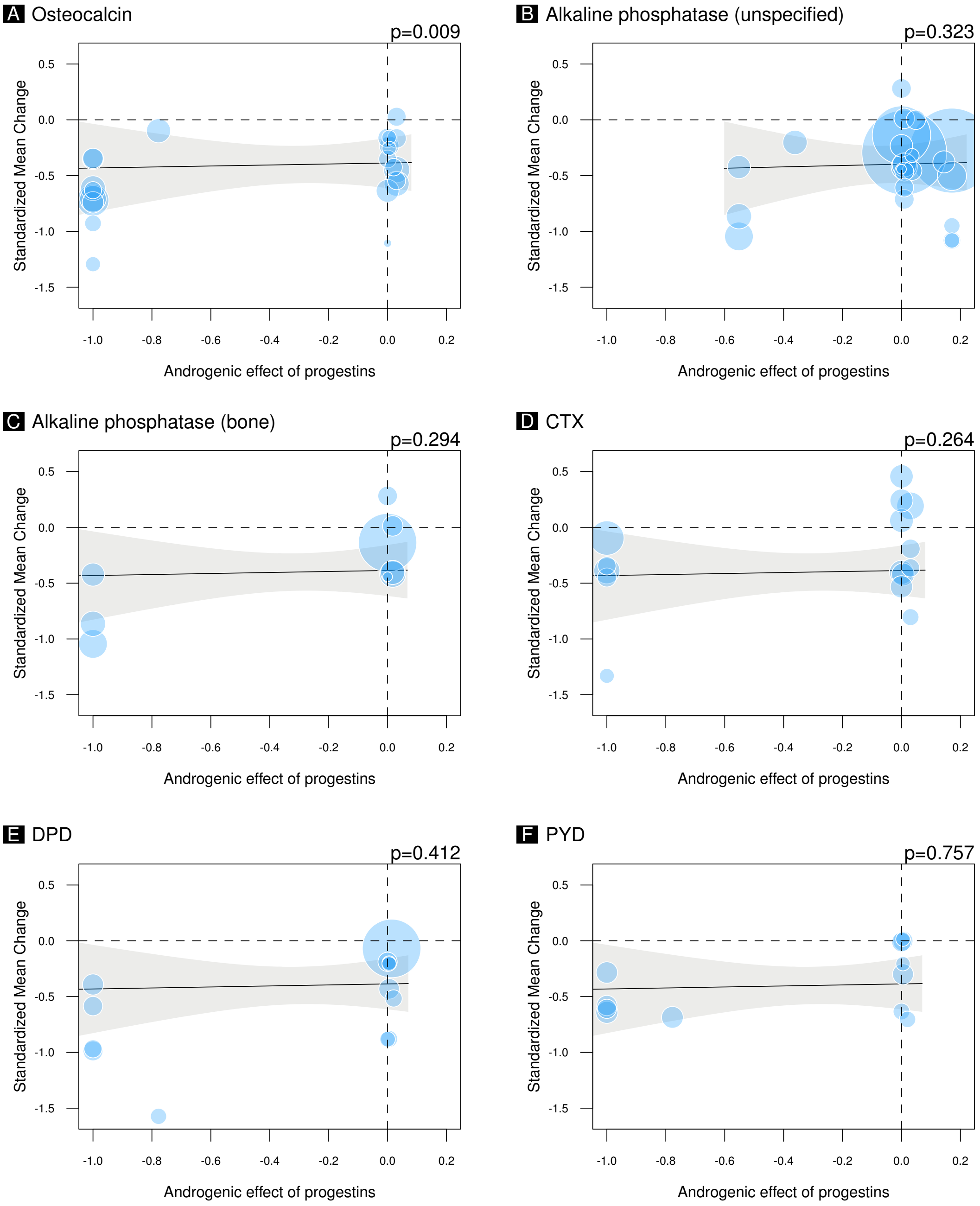

Figure S8- The illustration displays the forest plots of urine and serum calcium in a cohort of healthy women. Panel A shows the calcium (urine) SMC levels in healthy women before and after hormonal contraception. Panel B shows the levels of calcium (serum) SMC in healthy women before and after hormonal contraception. (*) Maximum age of women enrolled ≤ 21.

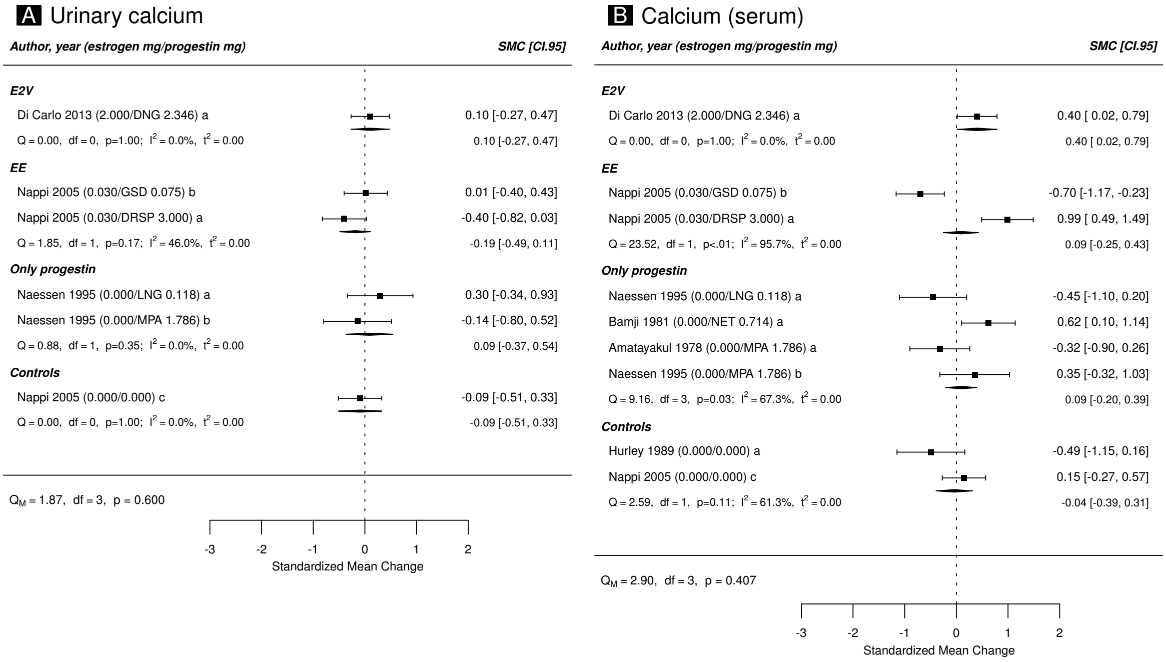

Figure S9- The provided figure depicts the forest plots representing the bone mass density (BMD) assessment in different anatomical locations of healthy women among the included studies: spine (Panel A), forearm (Panel B), total body (Panel C), total hip (Panel D), and femur (Panel E). (*) Maximum age of women enrolled $\leq$ 21.

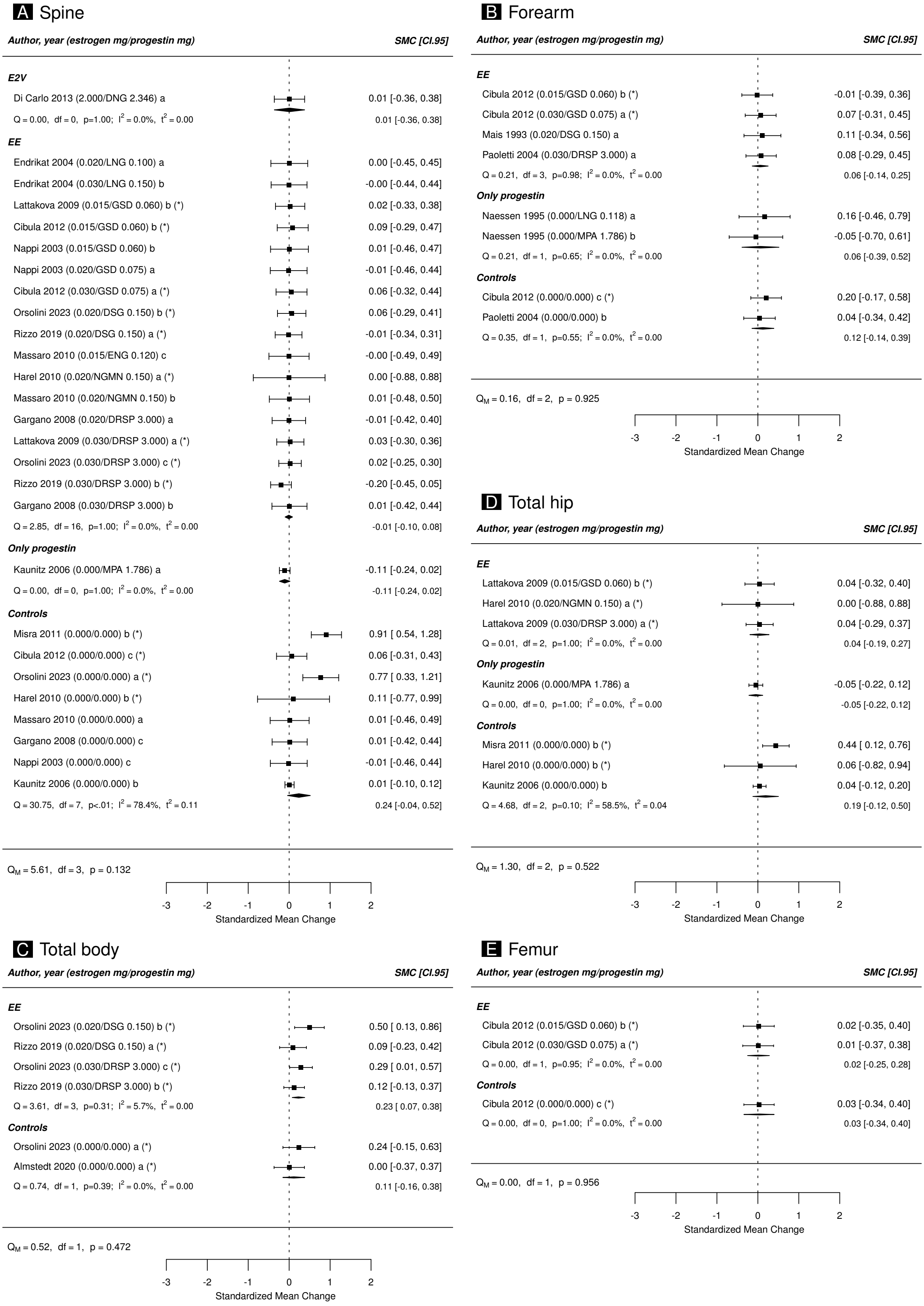

Figure S10- The figures illustrate the results of univariate meta-regressions of bone mass density (BMD) as the dependent variable. Panels A and B depict the outcomes of univariate meta-regressions investigating the association between the androgenic effects of progestins and their correlation with bone mineral density (BMD) in the spine (Panel A) and forearm (Panel B) when utilized in conjunction with estrogens for the treatment of healthy women. Panels C and D depict the outcomes of univariate meta-regressions investigating the link between estrogenic effect on sex hormone-binding globulin (SHBG) and bone mineral density (BMD) in the spine (Panel C) and forearm (Panel D) when estrogens are administered to healthy women. Panels E and F illustrate the outcomes of univariate meta-regressions investigating the link between estrogenic effect on sex hormone-binding globulin (SHBG) and bone mineral density (BMD) in the spine (Panel E) and forearm (Panel F) when estrogens (considering only EE) are administered to healthy women.

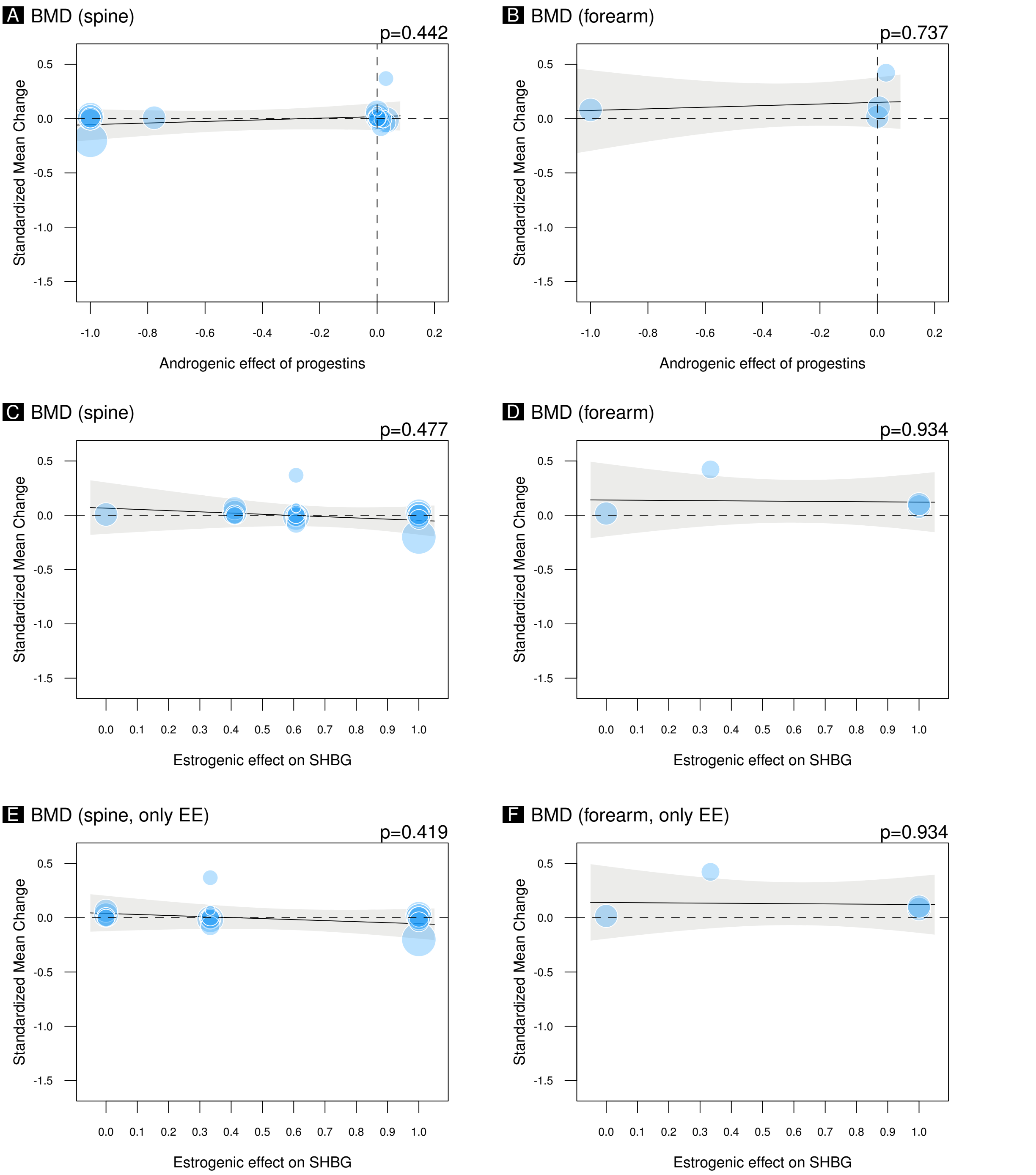

Figure S11- The visual representation illustrates the funnel plots and Egger's test results (shown p-values), indicating possible publication bias and the robustness of the meta-analysis conclusions. These panels show, in healthy women, osteocalcin (panel A), alkaline phosphatase (AP) all types unspecified and bone-specific (panel B), only bone-specific AP (panel C), P1NP (panel D), DPD (panel E), PYD (panel F), CTX (panel G), NTX (panel H), calcium (urine) (panel I), calcium (serum) (panel J), spine bone mineral density (BMD) (panel K), forearm BMD (panel L), total body BMD (panel M), total hip BMD (panel N), and femur BMD (panel O).

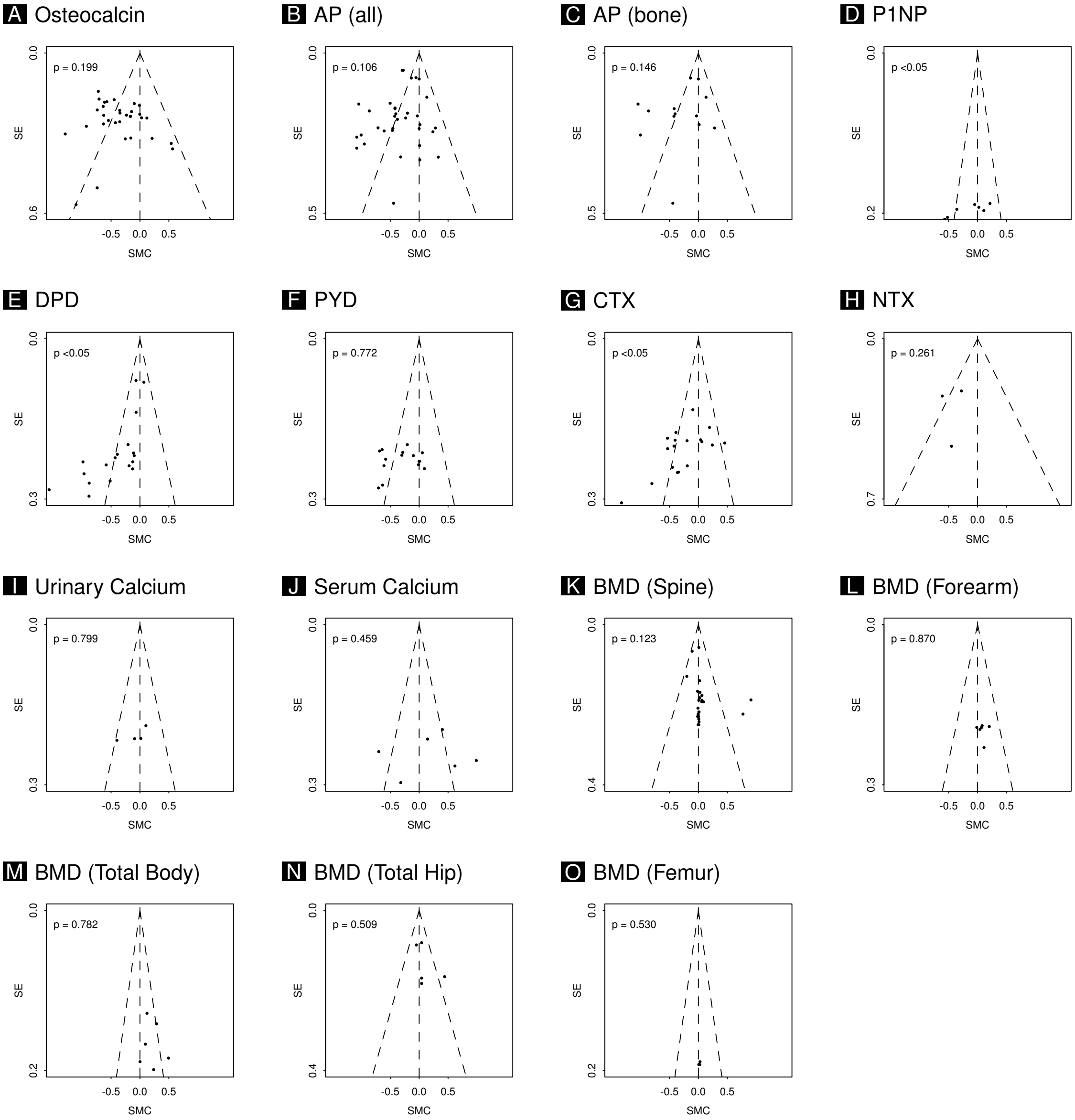

Table S1- The purpose of these points is to provide a concise overview of the objectives pursued in the study. The primary objective was developed utilizing the population intervention comparator outcome (PICO) framework.

N.	**Primary question**
1	What is the effect in terms of changes in osteocalcin levels in healthy women of childbearing age when comparing controls and various combinations of estrogen-progestogen therapy, including those preparations with natural estrogens?
	**Secondary questions**
2	What is the effect in terms of CTX, P1NP, NTX, PYD, DPD, calcium, ALP, sclerostin, and TRAcP 5b levels in healthy women of childbearing age, when comparing controls and various combinations of estrogen-progestogen therapy, including those with natural estrogens?
3	What estrogen-progestogen therapy-related factors influence bone markers in patients of childbearing age when comparing controls and various combinations of estrogen-progestogen therapy, including those preparations with natural estrogens?

Table S2- Included and excluded studies, with reasons.

Study	Included	Reason for exlusion
**INCLUDED STUDIES**		
Caldeirão TD, Orsolini LR, da Silva CC, Bisi Rizzo AdC, Teixeira AS, Nunes HRdC, Goldberg TBL. Effect of Two Combinations of Low-Dose Oral Contraceptives on Adolescent Bone Mass: A Clinical Trial with 2 Years Follow-Up. Medicine (Baltimore). 2022;101:e30680.	Yes	---
Almstedt HC, Cook MM, Bramble LF, Dabir DV, LaBrie JW. Oral Contraceptive Use, Bone Mineral Density, and Bone Turnover Markers over 12 Months in College-Aged Females. J Bone Miner Metab. 2020;38:544–554.	Yes	---
Tiedeken M, Westhoff CL, Cohen A, Cremers S, Sitruk-Ware R, Blithe DL, NICHD Contraceptive Trials Network Vaginal Ring Group. Bone Turnover Markers in Women Participating in a Dose-Finding Trial of a Contraceptive Vaginal Ring Releasing Nestorone and Estradiol. Contraception. 2019;99:329–334.	Yes	---
Rizzo AdCB, Goldberg TBL, Biason TP, Kurokawa CS, da Silva CC, Corrente JE, Nunes HRC. One-Year Adolescent Bone Mineral Density and Bone Formation Marker Changes through the Use or Lack of Use of Combined Hormonal Contraceptives. J Pediatr (Rio J). 2019;95:567–574.	Yes	---
Iltemir Duvan C, Onaran Y, Aktepe Keskin E, Yüce E, Yanık B, Kafali H, Ozturk Turhan N. Effects of the Etonogestrel Contraceptive Implant (Implanon®) on Bone Metabolism during Lactation: A Prospective Study. J Fam Plann Reprod Health Care. 2017;43:113–117.	Yes	---
Mawet M, Maillard C, Klipping C, Zimmerman Y, Foidart JM, Coelingh Bennink HJT. Unique Effects on Hepatic Function, Lipid Metabolism, Bone and Growth Endocrine Parameters of Estetrol in Combined Oral Contraceptives. Eur J Contracept Reprod Health Care. 2015;20:463–475.	Yes	---
Di Carlo C, Gargano V, Sparice S, Tommaselli GA, Bifulco G, Schettino D, Nappi C. Short-Term Effects of an Oral Contraceptive Containing Oestradiol Valerate and Dienogest on Bone Metabolism and Bone Mineral Density: An Observational, Preliminary Study. Eur J Contracept Reprod Health Care. 2013;18:388–393.	Yes	---
Massaro M, Di Carlo C, Gargano V, Formisano C, Bifulco G, Nappi C. Effects of the Contraceptive Patch and the Vaginal Ring on Bone Metabolism and Bone Mineral Density: A Prospective, Controlled, Randomized Study. Contraception. 2010;81:209–214.	Yes	---
Harel Z, Riggs S, Vaz R, Flanagan P, Harel D, Machan JT. Bone Accretion in Adolescents Using the Combined Estrogen and Progestin Transdermal Contraceptive Method Ortho Evra: A Pilot Study. J Pediatr Adolesc Gynecol. 2010;23:23–31.	Yes	---
Lattakova M, Borovsky M, Payer J, Killinger Z. Oral Contraception Usage in Relation to Bone Mineral Density and Bone Turnover in Adolescent Girls. Eur J Contracept Reprod Health Care. 2009;14:207–214.	Yes	---
Gargano V, Massaro M, Morra I, Formisano C, Di Carlo C, Nappi C. Effects of Two Low-Dose Combined Oral Contraceptives Containing Drospirenone on Bone Turnover and Bone Mineral Density in Young Fertile Women: A Prospective Controlled Randomized Study. Contraception. 2008;78:10–15.	Yes	---
Nappi C, Di Spiezio Sardo A, Greco E, Tommaselli GA, Giordano E, Guida M. Effects of an Oral Contraceptive Containing Drospirenone on Bone Turnover and	Yes	---

Study	Included	Reason for exlusion
Bone Mineral Density. Obstet Gynecol. 2005; 105:53–60.		
Rome E, Ziegler J, Secic M, Bonny A, Stager M, Lazebnik R, Cromer BA. Bone Biochemical Markers in Adolescent Girls Using Either Depot Medroxyprogesterone Acetate or an Oral Contraceptive. J Pediatr Adolesc Gynecol. 2004;17:373–377.	Yes	---
Paoletti AM, Orrù M, Lello S, Floris S, Ranuzzi F, Etzi R, Zedda P, Guerriero S, Fratta S, Sorge R, Mallarini G, Melis GB. Short-Term Variations in Bone Remodeling Markers of an Oral Contraception Formulation Containing 3 Mg of Drospirenone plus 30 Microg of Ethinyl Estradiol: Observational Study in Young Postadolescent Women. Contraception. 2004;70:293–298.	Yes	---
Endrikat J, Mih E, Düsterberg B, Land K, Gerlinger C, Schmidt W, Felsenberg D. A 3-Year Double-Blind, Randomized, Controlled Study on the Influence of Two Oral Contraceptives Containing Either 20 Microg or 30 Microg Ethinylestradiol in Combination with Levonorgestrel on Bone Mineral Density. Contraception. 2004; 69:179–187.	Yes	---
Nappi C, Di Spiezio Sardo A, Acunzo G, Bifulco G, Tommaselli GA, Guida M, Di Carlo C. Effects of a Low-Dose and Ultra-Low-Dose Combined Oral Contraceptive Use on Bone Turnover and Bone Mineral Density in Young Fertile Women: A Prospective Controlled Randomized Study. Contraception. 2003;67:355–359.	Yes	---
Paoletti AM, Orrù M, Floris S, Mannias M, Vacca AM, Ajossa S, Guerriero S, Melis GB. Evidence That Treatment with Monophasic Oral Contraceptive Formulations Containing Ethinylestradiol plus Gestodene Reduces Bone Resorption in Young Women. Contraception. 2000;61:259–263.	Yes	---
Naessen T, Olsson SE, Gudmundson J. Differential Effects on Bone Density of Progestogen-Only Methods for Contraception in Premenopausal Women. Contraception. 1995;52:35–39.	Yes	---
Mais V, Fruzzetti F, Ajossa S, Paoletti AM, Guerriero S, Melis GB. Bone Metabolism in Young Women Taking a Monophasic Pill Containing 20 Mcg Ethinylestradiol: A Prospective Study. Contraception. 1993;48:445–452.	Yes	---
Roy S, Mishell DR, Gray G, Dozono-Takano R, Brenner PF, Eide I, de Quattro V, Shaw ST. Comparison of Metabolic and Clinical Effects of Four Oral Contraceptive Formulations and a Contraceptive Vaginal Ring. Am J Obstet Gynecol. 1980;136:920–931.	Yes	---
Sadik W, Kovacs L, Pretnar-Darovec A, Mateo de Acosta O, Toddywalla VS, Dhall GI, Ng CSA, Holck S, Belsey E, Pinol A, Hall PE. A Randomized Double-Blind Study of the Effects of Two Low-Dose Combined Oral Contraceptives on Biochemical Aspects. Report from a Seven-Centred Study. WHO Special Programme of Research, Development and Research Training in Human Reproduction. Task Force on Oral Contraceptives. Contraception. 1985;32:223–236.	Yes	---
Cibula D, Skrenkova J, Hill M, Stepan JJ. Low-Dose Estrogen Combined Oral Contraceptives May Negatively Influence Physiological Bone Mineral Density Acquisition during Adolescence. Eur J Endocrinol. 2012;166:1003–1011.	Yes	---
Kaunitz AM, Miller PD, Rice VM, Ross D, McClung MR. Bone Mineral Density in Women Aged 25-35 Years Receiving Depot Medroxyprogesterone Acetate: Recovery Following Discontinuation. Contraception. 2006; 74:90–99.	Yes	---
Ahrén T, Victor A, Lithell H, Johansson ED. Comparison of the Metabolic Effects of Two Hormonal Contraceptive Methods: An Oral Formulation and a Vaginal	Yes	---

**Study**	**Included**	**Reason for exlusion**
Ring. I. Carbohydrate Metabolism and Liver Function. Contraception. 1981;24:415–427.		
Amatayakul K, Sivassomboon B, Singkamani R. Effects of Medroxyprogesterone Acetate on Serum Lipids, Protein, Glucose Tolerance and Liver Function in Thai Women. Contraception. 1980;21:283–297.	Yes	---
Hurley DL, Tiegs RD, Barta J, Laakso K, Heath H. Effects of Oral Contraceptive and Estrogen Administration on Plasma Calcitonin in Pre- and Postmenopausal Women. J Bone Miner Res. 1989;4:89–95.	Yes	---
Etzrodt R, Klinger G, Carol W. [The action of Sequostat in comparison to Sequence Ovosiston on selected metabolic parameters]. Zentralbl Gynakol. 1990;112:489–496.	Yes	---
García CR, Wallach EE. Liver Function Studies and Progestagen Contraception. Review of an Intramuscularly Administered Contraceptive. Fertil Steril. 1968;19:172–185.	Yes	---
Brügmann E, Göretzlehner G, Töwe J, Rehpenning W. [Liver function tests after a 6-month deposiston therapy]. Zentralbl Gynakol. 1975;97:669–673.	Yes	---
Bamji MS, Safaya S, Prema K. Low Dose Injectable Contraceptive Norethisterone Enanthate 20mg Monthly - II. Metabolic Side Effects. Contraception. 1981;23:23–36.	Yes	---
Hernandez-Juarez J, Garcia-Latorre EA, Moreno-Hernandez M, Moran-Perez JF, Rodriguez-Escobedo MA, Cogque-Hernandez G, Julián-Nacer R, Hernandez-Giron X, Palafox-Gomez R, Isordia-Salas I, Majluf-Cruz A. Metabolic Effects of the Contraceptive Skin Patch and Subdermal Contraceptive Implant in Mexican Women: A Prospective Study. Reprod Health. 2014;11:33.	Yes	---
Orsolini LR, Goldberg TBL, Caldeirão TD, Cristiane da Silva C, Rizzo AdCB, Biason TP, Teixeira AS, Carvalho Nunes HR. Bone Impact after Two Years of Low-Dose Oral Contraceptive Use during Adolescence. PLoS One. 2023;18:e0285885.	Yes	---
Amatayakul K, Sivasomboon B, Thanangkul O. Vitamin and Trace Mineral Metabolism in Medroxyprogesterone Acetate Users. Contraception. 1978;18:253–269.	Yes	---
Misra M, Katzman D, Miller KK, Mendes N, Snelgrove D, Russell M, Goldstein MA, Ebrahimi S, Clauss L, Weigel T, Mickley D, Schoenfeld DA, Herzog DB, Klibanski A. Physiologic Estrogen Replacement Increases Bone Density in Adolescent Girls with Anorexia Nervosa. J Bone Miner Res. 2011;26:2430–2438.	Yes	---
**EXCLUDED STUDIES**		
Cibula D, Stepan J, Skrenkova J, Hill M. BMD and Biochemical Markers of Bone Turnover in Adolescent Girls on Oral Contraceptives with Different Estrogen Content. Bone. 2009;44:S259.	No	Congress abstract
Caird LE, Reid-Thomas V, Hannan WJ, Gow S, Glasier AF. Oral Progestogen-Only Contraception May Protect against Loss of Bone Mass in Breast-Feeding Women. Clin Endocrinol (Oxf). 1994;41:739–745.	No	No exposure of interest
Svedlund A, Pettersson C, Tubic B, Ellegård L, Elfvin A, Magnusson P, Swolin-Eide D. Bone Mass and Biomarkers in Young Women with Anorexia Nervosa: A Prospective 3-Year Follow-up Study. J Bone Miner Metab. 2022;40:974–989.	No	No exposure of interest
Carmina E, Janni A, Lobo RA. Physiological Estrogen Replacement May Enhance the Effectiveness of the Gonadotropin-Releasing Hormone Agonist in the Treatment of Hirsutism. J Clin Endocrinol Metab. 1994; 78:126–130.	No	No exposure of interest

Study	Included	Reason for exlusion
Polatti F, Perotti F, Filippa N, Gallina D, Nappi RE. Bone Mass and Long-Term Monophasic Oral Contraceptive Treatment in Young Women. Contraception. 1995;51:221–224.	No	No outcomes of interest
Karlsson R, Eden S, von Schoultz B. Oral Contraception Affects Osteocalcin Serum Profiles in Young Women. Osteoporos Int. 1992;2:118–121.	No	No outcomes of interest
Sammour MB, Hilal SO. Alkaline Phosphatase Activity of Polymorphonulcear Leukocytes in Relation to Oral Contraceptives. Am J Obstet Gynecol. 1969;103:823–827.	No	No outcomes of interest
DeMerre LJ, Litofsky FS. Alkaline-Phosphatase Activity during Menstruation. Fertil Steril. 1968;19:593–597.	No	No outcomes of interest
Sall ND, Sow A, Toure M, Sarr GN, Seck I, Diadhiou F. [Biochemical profile of Senegalese women on oral contraceptives]. Dakar Med. 1992;37:159–162.	No	No outcomes of interest
Weijers MJ. Desogestrel, a New Progestational Compound, and the Liver. Arzneimittelforschung. 1983; 33:774–776.	No	No outcomes of interest
Cosson M, Querleu D, Donnez J, Madelenat P, Konincks P, Audebert A, Manhes H. Dienogest Is as Effective as Triptorelin in the Treatment of Endometriosis after Laparoscopic Surgery: Results of a Prospective, Multicenter, Randomized Study. Fertil Steril. 2002;77:684–692.	No	No outcomes of interest
Grant EC, Pryse-Davies J. Effect of Oral Contraceptives on Depressive Mood Changes and on Endometrial Monoamine Oxidase and Phosphatases. Br Med J. 1968;3:777–780.	No	No outcomes of interest
Abrahamsen B, Stilgren LS, Rettmer E, Bonnevie-Nielsen V, Beck-Nielsen H. Effects of the Natural and Artificial Menstrual Cycle on the Production of Osteoprotegerin and the Bone Resorptive Cytokines IL-1beta and IL-6. Calcif Tissue Int. 2003;72:18–23.	No	No outcomes of interest
Brocklehurst D, Wilde CE. Evidence for the Hormonal Regulation of the Multimolecular Forms of Serum Alkaline Phosphatase. Prog Clin Biol Res. 1984;166:277–288.	No	No outcomes of interest
Skouby SO. Laboratory and Clinical Assessment of a New Progestational Compound, Desogestrel: A Phase I Study. Acta Obstet Gynecol Scand. 1982;61:7–11.	No	No outcomes of interest
Brügmann E, Göretzlehner G, Klie E, Schwager A, Maass M. [Liver function studies under the effect of 4 sequential hormonal contraceptives]. Z Gesamte Inn Med. 1978;33:826–829.	No	No outcomes of interest
Brügmann E, Göretzlehner G, Dabels J, Töwe J. [Liver function studies under the influence of hormonal contraceptives (sequential preparations)]. Dtsch Z Verdau Stoffwechselkr. 1979;39:69–74.	No	No outcomes of interest
Akpowowo HE, Göretzlehner G, Brügmann E, Töwe J. [Liver function tests under the influence of sequential treatment using ethinyl estradiol-norethisterone acetate and ethinyl estradiol-chlormadinone acetate]. Zentralbl Gynakol. 1976;98:1198–1203.	No	No outcomes of interest
Larsson-Cohn U. Oral Contraception and Liver-Function Tests. Br Med J. 1965;1:1414–1415.	No	No outcomes of interest
Brügmann E, Göretzlehner G, Dabels J, Töwe J. Studies on Liver Function under the Influence of Oral Contraceptives. Int J Gynaecol Obstet. 1979;16:394–397.	No	No outcomes of interest
Schenker JH, Jungreis E, Polishuk WZ. Oral Contraceptives and Serum Copper Concentration. Obstet Gynecol. 1971;37:233–237.	No	No outcomes of interest
Klibanski A, Biller BM, Schoenfeld DA, Herzog DB, Saxe VC. The Effects of	No	No outcomes of

Study	Included	Reason for exlusion
Estrogen Administration on Trabecular Bone Loss in Young Women with Anorexia Nervosa. J Clin Endocrinol Metab. 1995;80:898–904.		interest
Coombs CV, O'Leary TJ, Tang JCY, Fraser WD, Greeves JP. Hormonal Contraceptive Use, Bone Density and Biochemical Markers of Bone Metabolism in British Army Recruits. BMJ Mil Health. 2023;169:9–16.	No	No prospective study
Shaarawy M, El-Mallah SY, Seoudi S, Hassan M, Mohsen IA. Effects of the Long-Term Use of Depot Medroxyprogesterone Acetate as Hormonal Contraceptive on Bone Mineral Density and Biochemical Markers of Bone Remodeling. Contraception. 2006;74:297–302.	No	No prospective study
Vanderjagt DJ, Sagay AS, Imade GE, Farmer SE, Glew RH. Effect of Norplant Contraceptive on the Bones of Nigerian Women as Assessed by Quantitative Ultrasound and Serum Markers of Bone Turnover. Contraception. 2005;72:212–216.	No	No prospective study
Ott SM, Scholes D, LaCroix AZ, Ichikawa LE, Yoshida CK, Barlow WE. Effects of Contraceptive Use on Bone Biochemical Markers in Young Women. J Clin Endocrinol Metab. 2001;86:179–185.	No	No prospective study
Schiele F, Vincent-Viry M, Fournier B, Starck M, Siest G. Biological Effects of Eleven Combined Oral Contraceptives on Serum Triglycerides, Gamma-Glutamyltransferase, Alkaline Phosphatase, Bilirubin and Other Biochemical Variables. Clin Chem Lab Med. 1998;36:871–878.	No	No prospective study
Garnero P, Sornay-Rendu E, Delmas PD. Decreased Bone Turnover in Oral Contraceptive Users. Bone. 1995;16:499–503.	No	No prospective study
Ponthieux A, Herbeth B, Droesch S, Haddy N, Lambert D, Visvikis S. Biological Determinants of Serum ICAM-1, E-selectin, P-selectin and L-selectin Levels in Healthy Subjects: The Stanislas Study. Atherosclerosis. 2004;172:299–308.	No	No prospective study
Weinbrenner T, Zittermann A, Gouni-Berthold I, Stehle P, Berthold HK. Body Mass Index and Disease Duration Are Predictors of Disturbed Bone Turnover in Anorexia Nervosa. A Case-Control Study. Eur J Clin Nutr. 2003;57:1262–1267.	No	No prospective study
Callegari ET, Garland SM, Gorelik A, Chiang CY, Wark JD. Bone Turnover Marker Determinants in Young Women: Results from the Safe-D Study. Ann Clin Biochem. 2018;55:328–340.	No	No prospective study
Maïmoun L, Guillaume S, Lefebvre P, Philibert P, Bertet H, Picot MC, Gaspari L, Paris F, Seneque M, Dupuys AM, Courtet P, Thomas E, Mariano-Goulart D, Bringer J, Renard E, Sultan C. Evidence of a Link between Resting Energy Expenditure and Bone Remodelling, Glucose Homeostasis and Adipokine Variations in Adolescent Girls with Anorexia Nervosa. Osteoporos Int. 2016;27:135–146.	No	No prospective study
Campesi I, Sanna M, Zinellu A, Carru C, Rubattu L, Bulzomi P, Seghieri G, Tonolo G, Palermo M, Rosano G, Marino M, Franconi F. Oral Contraceptives Modify DNA Methylation and Monocyte-Derived Macrophage Function. Biol Sex Differ. 2012;3:4.	No	No prospective study
Guañabens N, Filella X, Monegal A, Gómez-Vaquero C, Bonet M, Buquet D, Casado E, Cerdá D, Erra A, Martinez S, Montalá N, Pitarch C, Kanterewicz E, Sala M, Surís X, Torres F, LabOscat Study Group. Reference Intervals for Bone Turnover Markers in Spanish Premenopausal Women. Clin Chem Lab Med. 2016; 54:293–303.	No	No prospective study
Revilla M, Fraile E, Aguado F, Hermandez ER, Villa LF, Rico H. Vertebral and Metacarpal Morphometry as Indicators of Nutritional Improvement. Clin Rheumatol. 1997;16:279–283.	No	No prospective study

Study	Included	Reason for exlusion
Simpson GR, Dale E. Serum Levels of Phosphorus, Magnesium, and Calcium in Women Utilizing Combination Oral or Long-Acting Injectable Progestational Contraceptives. Fertil Steril. 1972;23:326–330.	No	No prospective study
Seeman E, Szmukler GI, Formica C, Tsalamandris C, Mestrovic R. Osteoporosis in Anorexia Nervosa: The Influence of Peak Bone Density, Bone Loss, Oral Contraceptive Use, and Exercise. J Bone Miner Res. 1992; 7:1467–1474.	No	No prospective study
Engineer AD, Gupta V, Tandon P. Liver Function Tests in Patients on Oral Progestogens. J Obstet Gynaecol India. 1968;18:598–605.	No	No separated data for specific exposure
Chieffi O, Brogioni M, Saltarelli O, Pecchioli S, Benardi M. Oestroprogestogen interference on some haematochemical parameters: glycaemia, lipid metabolism and hepatic functionality (author's transl). Patol Clin Ostet Ginecol. 1981;9:7–17.	No	No separated data for specific exposure
Guisado-Cuadrado I, Romero-Parra N, Elliott-Sale KJ, Sale C, Díaz ÁE, Peinado AB. Influence of Menstrual Cycle and Oral Contraceptive Phases on Bone (Re)Modelling Markers in Response to Interval Running. Calcif Tissue Int. 2024;115:382–392.	No	No separated data for specific exposure
DeMasi T, Tsang M, Mueller J, Giltvedt K, Nguyen TN, Kern M, Hooshmand S. Prunes May Blunt Adverse Effects of Oral Contraceptives on Bone Health in Young Adult Women: A Randomized Clinical Trial. Curr Dev Nutr. 2024;8:104417.	No	No separated data for specific exposure
Donangelo CM, Cornes R, Sintes C, Bezerra FF. Combined Oral Contraceptives: Association with Serum 25-Hydroxyvitamin D and Calcium and Bone Homeostasis. J Womens Health (Larchmt). 2024;33:805–815.	No	No separated data for specific exposure
Resulaj M, Polineni S, Meenaghan E, Eddy K, Lee H, Fazeli PK. Transdermal Estrogen in Women With Anorexia Nervosa: An Exploratory Pilot Study. JBMR Plus. 2020;4:e10251.	No	No target population
Singhal V, Ackerman KE, Bose A, Flores LPT, Lee H, Misra M. Impact of Route of Estrogen Administration on Bone Turnover Markers in Oligoamenorrheic Athletes and Its Mediators. J Clin Endocrinol Metab. 2019; 104:1449–1458.	No	No target population
Warren MP, Miller KK, Olson WH, Grinspoon SK, Friedman AJ. Effects of an Oral Contraceptive (Norgestimate/Ethinyl Estradiol) on Bone Mineral Density in Women with Hypothalamic Amenorrhea and Osteopenia: An Open-Label Extension of a Double-Blind, Placebo-Controlled Study. Contraception. 2005; 72:206–211.	No	No target population
Grinspoon SK, Friedman AJ, Miller KK, Lippman J, Olson WH, Warren MP. Effects of a Triphasic Combination Oral Contraceptive Containing Norgestimate/Ethinyl Estradiol on Biochemical Markers of Bone Metabolism in Young Women with Osteopenia Secondary to Hypothalamic Amenorrhea. J Clin Endocrinol Metab. 2003;88:3651–3656.	No	No target population
Castelo-Branco C, Vicente JJ, Pons F, Martínez de Osaba MJ, Casals E, Vanrell JA. Bone Mineral Density in Young, Hypothalamic Oligoamenorrheic Women Treated with Oral Contraceptives. J Reprod Med. 2001; 46:875–879.	No	No target population
Gregoriou O, Bakas P, Konidaris S, Papadias K, Mathiopoulos D, Creatsas G. The Effect of Combined Oral Contraception with or without Spironolactone on Bone Mineral Density of Hyperandrogenic Women. Gynecol Endocrinol. 2000;14:369–373.	No	No target population
Volpe A, Amram A, Cagnacci A, Battaglia C. Biochemical Aspects of Hormonal Contraception: Effects on Bone Metabolism. Eur J Contracept Reprod Health Care. 1997;2:123–126.	No	No target population

Study	Included	Reason for exlusion
Ackerman KE, Singhal V, Baskaran C, Slattery M, Campoverde Reyes KJ, Toth A, Eddy KT, Bouxsein ML, Lee H, Klibanski A, Misra M. Oestrogen Replacement Improves Bone Mineral Density in Oligo-Amenorrhoeic Athletes: A Randomised Clinical Trial. Br J Sports Med. 2019;53:229–236.	No	No target population
Tuppurainen M, Kröger H, Saarikoski S, Honkanen R, Alhava E. The Effect of Previous Oral Contraceptive Use on Bone Mineral Density in Perimenopausal Women. Osteoporos Int. 1994;4:93–98.	No	No target population
Bergmann P, Valsamis J, Van Perborgh J, De Schepper J, Van Vliet G. Comparative Study of the Changes in Insulin-like Growth Factor-I, Procollagen-III N-terminal Extension Peptide, Bone Gla-protein, and Bone Mineral Content in Children with Turner’s Syndrome Treated with Recombinant Growth Hormone. J Clin Endocrinol Metab. 1990;71:1461–1467.	No	No target population
Suwikrom S, Jaisamrarn U. Comparison of the Metabolic Effects of Oral Contraceptive and Nonhormonal Contraceptive Use in Women over 40 Years Old. Contraception. 2005;71:183–187.	No	No target population
Grecu EO, Weinshelbaum A, Simmons R. Effective Therapy of Glucocorticoid-Induced Osteoporosis with Medroxyprogesterone Acetate. Calcif Tissue Int. 1990;46:294–299.	No	No target population
DiVasta AD, Feldman HA, O’Donnell JM, Long J, Leonard MB, Gordon CM. Impact of Adrenal Hormone Supplementation on Bone Geometry in Growing Teens With Anorexia Nervosa. J Adolesc Health. 2019; 65:462–468.	No	No target population
Falsetti L, Galbignani E. Long-Term Treatment with the Combination Ethinylestradiol and Cyproterone Acetate in Polycystic Ovary Syndrome. Contraception. 1990;42:611–619.	No	No target population
Gambacciani M, Ciaponi M, Cappagli B, Benussi C, Genazzani AR. Longitudinal Evaluation of Perimenopausal Femoral Bone Loss: Effects of a Low-Dose Oral Contraceptive Preparation on Bone Mineral Density and Metabolism. Osteoporos Int. 2000;11:544–548.	No	No target population
Gambacciani M, Spinetti A, Taponeco F, Cappagli B, Piaggesi L, Fioretti P. Longitudinal Evaluation of Perimenopausal Vertebral Bone Loss: Effects of a Low-Dose Oral Contraceptive Preparation on Bone Mineral Density and Metabolism. Obstet Gynecol. 1994;83:392–396.	No	No target population
Vexiau P, Gueux B, Vexiau-Robert D, Fiet J, Laureaux C, Tabuteau F, Brerault JL, Mathieson J, Cathelineau G. Metabolic Effects of Combined Cyproterone Acetate and Percutaneous 17 Beta Oestradiol after Six and Twelve Months Therapy in 61 Patients. Horm Metab Res. 1988;20:765–769.	No	No target population
Divasta AD, Feldman HA, Giancaterino C, Rosen CJ, Leboff MS, Gordon CM. The Effect of Gonadal and Adrenal Steroid Therapy on Skeletal Health in Adolescents and Young Women with Anorexia Nervosa. Metabolism. 2012;61:1010–1020.	No	No target population
Gordon CM, Grace E, Emans SJ, Feldman HA, Goodman E, Becker KA, Rosen CJ, Gundberg CM, LeBoff MS. Effects of Oral Dehydroepiandrosterone on Bone Density in Young Women with Anorexia Nervosa: A Randomized Trial. J Clin Endocrinol Metab. 2002;87:4935–4941.	No	No target population
Gordon CM, Grace E, Emans SJ, Goodman E, Crawford MH, Leboff MS. Changes in Bone Turnover Markers and Menstrual Function after Short-Term Oral DHEA in Young Women with Anorexia Nervosa. J Bone Miner Res. 1999;14:136–145.	No	No target population
Maharjan AS, Wyness SP, Ray JA, Willcox TL, Seiter JD, Genzen JR. Detection	No	Non human

Study	Included	Reason for exlusion
and Characterization of Estradiol (E2) and Unconjugated Estriol (uE3) Immunoassay Interference Due to Anti-Bovine Alkaline Phosphatase (ALP) Antibodies. Pract Lab Med. 2019;17:e00131.		
Iida J, Yoshikawa T, Miyazaki K, Okumura N, Takakura Y. Osteogenic Potential of Estriol-Treated Cultured Bone - In Vitro and In Vivo -. KEM. 2005;284–286:651–654.	No	Non human
Douxfils J, Gaspard U, Taziaux M, Jost M, Bouvy C, Lobo RA, Utian WH, Foidart JM. Impact of Estetrol (E4) on Hemostasis, Metabolism and Bone Turnover in Postmenopausal Women. Climacteric. 2023;26:55–63.	No	Post-menopausal
Costa-Paiva L, O Wender MC, Machado RB, Pompei LM, Nahas EA, Nahas-Neto J, Del Debbio SY, Badalotti M, Cruz AM. Effects of Ultra-Low Dose Hormone Therapy on Biochemical Bone Turnover Markers in Postmenopausal Women: A Randomized, Placebo-Controlled, Double-Blind Trial. Post Reprod Health. 2022; 28:149–157.	No	Post-menopausal
Christiansen C. Effects of Drospirenone/Estrogen Combinations on Bone Metabolism. Climacteric. 2005;8 Suppl 3:35–41.	No	Post-menopausal
Nguyên-Pascal ML, Thomas JL, Bergougnoux L, Garnero P, Drapier-Faure E, Delmas PD. Nomegestrol Acetate May Enhance the Skeletal Effects of Estradiol on Biochemical Markers of Bone Turnover in Menopausal Women after a 12-Week Treatment Period. Climacteric. 2005;8:136–145.	No	Post-menopausal
Ormarsdóttir S, Mallmin H, Naessén T, Petrén-Mallmin M, Broomé U, Hultcrantz R, Lööf L. An Open, Randomized, Controlled Study of Transdermal Hormone Replacement Therapy on the Rate of Bone Loss in Primary Biliary Cirrhosis. J Intern Med. 2004;256:63–69.	No	Post-menopausal
Gutteridge DH, Holzherr ML, Retallack RW, Price RI, Will RK, Dhaliwal SS, Faulkner DL, Stewart GO, Stuckey BGA, Prince RL, Criddle RA, Drury PJ, Tran L, Bhagat CI, Kent GN, Jamrozik K. A Randomized Trial Comparing Hormone Replacement Therapy (HRT) and HRT plus Calcitriol in the Treatment of Postmenopausal Osteoporosis with Vertebral Fractures: Benefit of the Combination on Total Body and Hip Density. Calcif Tissue Int. 2003;73:33–43.	No	Post-menopausal
Paoletti AM, Pilloni M, Orrù M, Floris S, Pistis M, Guerriero S, Ajossa S, Melis GB. Efficacy and Safety of Oral and Transdermal Hormonal Replacement Treatment Containing Levonorgestrel. Maturitas. 2002;42:137–147.	No	Post-menopausal
Bernard-Poenaru O, Roux C, Blanqué R, Gardner C, de Vemejoul MC, Cohen-Solal ME. Bone-Resorbing Cytokines from Peripheral Blood Mononuclear Cells after Hormone Replacement Therapy: A Longitudinal Study. Osteoporos Int. 2001;12:769–776.	No	Post-menopausal
Yoshitake K, Yokota K, Kasugai Y, Kagawa M, Sukamoto T, Nakamura T. Effects of 16 Weeks of Treatment with Tibolone on Bone Mass and Bone Mechanical and Histomorphometric Indices in Mature Ovariectomized Rats with Established Osteopenia on a Low-Calcium Diet. Bone. 1999;25:311–319.	No	Post-menopausal
Rogers A, Eastell R. Effects of Estrogen Therapy of Postmenopausal Women on Cytokines Measured in Peripheral Blood. J Bone Miner Res. 1998;13:1577–1586.	No	Post-menopausal
Prelević GM, Beljić T, Balint-Perić L, Petrović J, Elliesen J. Effect of Two Different Progestins (Cyproterone Acetate and Norgestrel), Administered in a Cyclical Estradiol Valerate Regimen, on Markers of Bone Turnover. Gynecol Endocrinol. 1994;8:209–214.	No	Post-menopausal
Saure A, Hirvonen E, Tikkanen MJ, Viinikka L, Ylikorkala O. A Novel Oestradiol–Desogestrel Preparation for Hormone Replacement Therapy: Effects	No	Post-menopausal

Study	Included	Reason for exlusion
on Hormones, Lipids, Bone, Climacteric Symptoms and Endometrium. Maturitas. 1993;16:1–12.		
Tarallo P, Henny J, Fournier B, Siest G. Plasma Osteocalcin: Biological Variations and Reference Limits. Scand J Clin Lab Invest. 1990;50:649–655.	No	Post-menopausal
Leis D, Zach H, Kohler P. [Comparative high rising dose study of oral 17-alpha-ethinylestradiol (EE2), estriol (E3), and parenteral 16-alpha-17-beta-estrioldihemisuccinate (E3-suc) in their effects on serum levels of glutamate transaminase (GOT), pyruvate transaminase (GPT), leucine amino peptidase (LAP), alkaline phosphatase (AP), and bilirubin in 30 hysterectomized and ovarectomized women (author's transl)]. Arch Gynecol. 1978;226:333–339.	No	Post-menopausal
Marslew U, Riis BJ, Christiansen C. Desogestrel in Hormone Replacement Therapy: Long-Term Effects on Bone, Calcium and Lipid Metabolism, Climacteric Symptoms, and Bleeding. Eur J Clin Invest. 1991;21:601–607.	No	Post-menopausal
Agostino H, Di Meglio G. Low-Dose Oral Contraceptives in Adolescents: How Low Can You Go? J Pediatr Adolesc Gynecol. 2010;23:195–201.	No	Review
Herrmann M, Seibel MJ. The Effects of Hormonal Contraceptives on Bone Turnover Markers and Bone Health. Clin Endocrinol (Oxf). 2010;72:571–583.	No	Review
Rocca ML, Palumbo AR, Bitonti G, Brisinda C, DI Carlo C. Bone Health and Hormonal Contraception. Minerva Obstet Gynecol. 2021;73:678–696.	No	Review
Wei S, Winzenberg T, Laslett LL, Venn A, Jones G. Oral Contraceptive Use and Bone. Curr Osteoporos Rep. 2011;9:6–11.	No	Review

Table S3- Presented below is a summary outlining the features of the arms included in the study.

Study	Arm	Formulation	Estrogens	Dose (mcg)	Progestins	Dose (mcg)	Number
Orsolini 2023	a	None	Controls	0	Controls	0	31
	b	Oral	EE	20	DSG	150	55
	c	Oral	EE	30	DRSP	3000	82
Caldeirão 2022	a	None	Controls	0	Controls	0	33
	b	Oral	EE	20	DSG	150	37
	c	Oral	EE	30	DRSP	3000	57
Almstedt 2020	a	None	Controls	0	Controls	0	28
Tiedeken 2019	a	Vaginal	E2	10	SGA	200	27
	b	Vaginal	E2	20	SGA	200	29
	c	Vaginal	E2	40	SGA	200	26
Rizzo 2019	a	Oral	EE	20	DSG	150	36
	b	Oral	EE	30	DRSP	3000	61
	c	None	Controls	0	Controls	0	70
Iltemir Duvan 2017	a	None	Controls	0	Controls	0	25
	b	Inplant	None	0	ENG	68000	25
Mawet 2015	a	Oral	E4	5000	DRSP	3000	17
	b	Oral	E4	10000	DRSP	3000	19
	c	Oral	EE	20	DRSP	3000	20
	d	Oral	E4	5000	LNG	150	18
	e	Oral	E4	10000	LNG	150	17
	f	Oral	E4	20000	LNG	150	18
Hernandez-Juarez 2014	a	Patch	EE	60	NGM	6000	25
	b	Inplant	None	0	ENG	68000	37
Di Carlo 2013	a	Oral	E2V	2000	DNG	2346	28
Cibula 2012	a	Oral	EE	30	GSD	75	27
	b	Oral	EE	15	GSD	60	27
	c	None	Controls	0	Controls	0	28
Misra 2011	b	None	Controls	0	Controls	0	40
Massaro 2010	a	None	Controls	0	Controls	0	17
	b	Patch	EE	20	NGMN	150	16
	c	Vaginal	EE	15	ENG	120	16
Harel 2010	a	Patch	EE	20	NGMN	150	5
	b	None	Controls	0	Controls	0	5
Lattakova 2009	a	Oral	EE	30	DRSP	3000	35
	b	Oral	EE	15	GSD	60	30
Gargano 2008	a	Oral	EE	20	DRSP	3000	23
	b	Oral	EE	30	DRSP	3000	21
	c	None	Controls	0	Controls	0	21
Kaunitz 2006	a	Depot	None	0	MPA	150000	248
	b	None	Controls	0	Controls	0	360
Nappi 2005	a	Oral	EE	30	DRSP	3000	23
	b	Oral	EE	30	GSD	75	22
	c	None	Controls	0	Controls	0	22
Paoletti 2004	a	Oral	EE	30	DRSP	3000	28
	b	None	Controls	0	Controls	0	26
Endrikat 2004	a	Oral	EE	20	LNG	100	19
	b	Oral	EE	30	LNG	150	20
Rome 2004	a	Depot	None	0	MPA	150000	53
	b	Oral	EE	20	LNG	100	165
	c	None	Controls	0	Controls	0	152
Nappi 2003	a	Oral	EE	20	GSD	75	19
	b	Oral	EE	15	GSD	60	18
	c	None	Controls	0	Controls	0	19

Paoletti 2000	a	Oral	EE	20	GSD	75	10
	b	Oral	EE	30	GSD	75	10
	c	Oral	Controls	0	Controls	0	10
Naessen 1995	a	Inplant	None	0	LNG	118	10
	b	Depot	None	0	MPA	150000	9
Mais 1993	a	Oral	EE	20	DSG	150	19
Etzrodt 1990	a	Oral	EE	50	NET	1000	46
	b	Oral	EEME	80	CMA	2000	29
Hurley 1989	a	None	Controls	0	Controls	0	10
Sadik 1985	a	Oral	EE	35	NET	1000	429
	b	Oral	EE	30	LNG	150	418
	c	Cu-IUD	Controls	0	Controls	0	195
Ahrén 1981	a	Vaginal	E2	180	LNG	290	22
	b	Oral	EE	30	LNG	150	20
Bamji 1981	a	Depot	None	0	NET	20000	17
Roy 1980	a	Oral	EEME	50	NET	1000	25
	b	Oral	EE	50	NET	1000	25
	c	Oral	EE	35	NET	1000	25
	d	Oral	EE	30	LNG	150	25
	e	Vaginal	E2	212	LNG	289	10
Amatayakul 1980	a	Depot	None	0	MPA	150000	12
Amatayakul 1978	a	Depot	None	0	MPA	150000	12
Brügmann 1975	a	Oral (depot effect)	EES	107	NET	357	18
García 1968	a	Depot	E2EN	10000	DHPA	150000	25

Table S4- Various biochemical markers are employed in studies on bone metabolism to evaluate the state of bone health and the rate at which bone is being remodeled. The bone resorption and formation markers assessed in this review are enumerated below.

Marker	Label	Description	Intrepretation
**Bone formation**			
Osteocalcin	OC	Calcium-binding peptide, released by mature osteoblasts, is a significant non-collagenous protein in bone. It is a recognized marker of bone development (increased during bone formation), but when incorporated into the organic bone matrix, it can also be released during osteoclastic bone resorption.	Increased with bone formation.
Alkaline phosphatase/bone-specific alkaline phosphatase	ALP/BAP	Reflects osteoblasts' number and differentiation state and provides information about bone anabolic activity. BAP is specific for the bone tissue.	Increased with bone formation.
N-terminal propeptide of procollagen type I	P1NP	A good marker of bone formation. Its insensitivity to circadian cycles and nutritional factors distinguishes it as a good tool in bone health assessment	Increased with bone formation.
**Bone resorption**			
C-terminal telopeptide	CTX	Fragment produced from the breakdown of type I collagen, the most prevalent protein in bone. During osteoclastic resorption, it is released into the bloodstream, and its amounts reflect the rate of bone resorption.	Increased with bone resorption.
N-terminal telopeptide	NTX	Fragment produced from the breakdown of type I collagen, the most prevalent protein in bone. During osteoclastic resorption, it is released into the bloodstream, and its amounts reflect the rate of bone resorption.	Increased with bone resorption.
Pyridinoline	PYD	Cross-link within structural collagens like bone and cartilage. PYD is primarily assessed in urine, free and peptide-bound, and rise with bone reabsorption.	Increased with bone resorption.
Deoxypyridinoline	DPD	Cross-link within structural collagens like bone and cartilage. DPD, predominantly present in bone, is a more specific marker of bone turnover than PYD. DPD is primarily assessed in urine, free and peptide-bound, and rise with bone reabsorption.	Increased with bone resorption.
Sclerostin	SOST	Primarily generated in bone tissue by mature osteocytes, is detectable in plasma, and helps us understand bone health because low levels are related to increased bone formation.	Increased with bone resorption.
Tartrate-resistant acid phosphatase 5b	TRACP 5b	Osteoclastic enzyme traditionally used to measure bone resorption. Specific immunoassays for the 5b isoform have recently been developed, providing insight into the quantity of osteoclasts. TRAcP5b is emerging as a potentially helpful marker for diseases marked by severe osteolysis	Increased with bone resorption.

For details see the following references [74–76, 79, 80]

Table S5- This table provides a concise overview of database queries.

Database	Query	Date	Number of items
PubMed	((Oral contraception) OR estriol OR (ethinyl estradiol) OR estetrol OR (hormonal contraceptive)) AND (osteocalcin OR (Alkaline phosphatase) OR APL OR CTX OR NTX OR Pyridinoline OR PYD OR Deoxy-Pyridinoline OR DPD OR (Tartrate-resistant acid phosphatase) OR (TRAcP 5b) OR P1NP)	11.12.2024	865
Scopus	TITLE-ABS-KEY(((Oral contraception) OR estriol OR (ethinyl estradiol) OR estetrol OR (hormonal contraceptive)) AND (osteocalcin OR (Alkaline phosphatase) OR APL OR CTX OR NTX OR Pyridinoline OR PYD OR Deoxy-Pyridinoline OR DPD OR (Tartrate-resistant acid phosphatase) OR (TRAcP 5b) OR P1NP))	11.12.2024	634
Cochrane Central Register of Controlled Trials and reviews	"Oral contraception" OR "estriol" OR "ethinyl estradiol" OR estetrol OR "hormonal contraceptive" AND osteocalcin OR "Alkaline phosphatase" OR APL OR CTX OR NTX OR Pyridinoline OR PYD OR Deoxy-Pyridinoline OR DPD OR "Tartrate-resistant acid phosphatase" OR "TRAcP 5b" OR P1NP	11.12.2024	131
EMBASE	AB,TI(((Oral contraception) OR estriol OR (ethinyl estradiol) OR estetrol OR (hormonal contraceptive)) AND (osteocalcin OR (Alkaline phosphatase) OR APL OR CTX OR NTX OR Pyridinoline OR PYD OR Deoxy-Pyridinoline OR DPD OR (Tartrate-resistant acid phosphatase) OR (TRAcP 5b) OR P1NP))	11.12.2024	258

Table S6- The andorgenic effect of progestogens.

Progestogen acronym	Progestogen name	Androgenic effect
LNG	Levonorgestrol	1
GSD	Gestodene	1
NET	Norethisterone	1
NGM	Norgestimate	1
DSG	Desogestrel	1
ENG	Etonogestrel	1
MPA	Medroxyprogesterone acetate	0.5
NGMN	Norelgestromin	0
Controls	No progestogen use	0
DHPA	Dihydroxyprogesterone acetophenide	0
SGA	Segesterone acetate	0
P4	Progesterone	-0.5
CMA	Chlormadinone acetate	-1
DRSP	Drospirenone	-1
DNG	Dienogest	-1
CPA	Cyproterone acetate	-2

The score was based on the following references and expert opinion [95–102].

Table S7- Relative potency refers to the comparative strength of a substance in relation to another. In this case, the effect of a 1mg dosage of estrogen on sex hormone-binding globulin (SHBG) levels is being assessed and compared to the potency of estradiol.

**Estrogens acronym**	**Estrogens name**	**Relative potency**
Controls	No estrogens use	0
E2	Estradiol	1
E2V	Estradiol valerate	1
E2-EN	Estradiol enanthate	1
E4	Estetrol	0.16
EE	Ethinylestradiol	443
EEME	Ethinylestradiol 3-methyl ether	443
EES	Ethinylestradiol sulfonate	443

The score was based on the following references and expert opinion [18, 88].
